# Jasmonic and salicylic acid pathways shape the rhizosphere microbiome, affecting aphid herbivory and soil-mediated insect–plant interactions in a density-dependent manner

**DOI:** 10.1093/pcp/pcag048

**Published:** 2026-04-14

**Authors:** Marcela Aragón, Haris Spyridis, Pieter Mostard, Michael Reichelt, Jonathan Gershenzon, Marcel Dicke, Karen J Kloth

**Affiliations:** Laboratory of Entomology, Wageningen University & Research, Droevendaalsesteeg 1, 6700 AA, Wageningen, The Netherlands; Laboratory of Entomology, Wageningen University & Research, Droevendaalsesteeg 1, 6700 AA, Wageningen, The Netherlands; Laboratory of Entomology, Wageningen University & Research, Droevendaalsesteeg 1, 6700 AA, Wageningen, The Netherlands; Department of Biochemistry, Max Planck Institute for Chemical Ecology, Hans-Knöll Straße 8, 07745, Jena, Germany; Department of Biochemistry, Max Planck Institute for Chemical Ecology, Hans-Knöll Straße 8, 07745, Jena, Germany; Laboratory of Entomology, Wageningen University & Research, Droevendaalsesteeg 1, 6700 AA, Wageningen, The Netherlands; Laboratory of Entomology, Wageningen University & Research, Droevendaalsesteeg 1, 6700 AA, Wageningen, The Netherlands

**Keywords:** plant–soil feedback, insect–plant interactions, phytohormone signaling, insect feeding guilds, induced systemic resistance

## Abstract

Aboveground induction of plant defense pathways can shape root-associated microbial communities. However, whether these changes are pathway-specific and how they affect plant growth and resistance remain unclear. We evaluated how induction of the jasmonic acid (JA) and salicylic acid (SA) defense pathways shapes the root microbiome of *Brassica oleracea* and whether these soil-mediated shifts affect plant growth and resistance to herbivory in a subsequent generation using a plant–soil feedback approach. In the conditioning phase, defense pathways were induced either through foliar application of methyl jasmonate and SA solutions or through herbivory by caterpillars (JA) and aphids (SA). Both pathways led to distinct shifts in microbial communities, with the bacterial and fungal composition varying by pathway identity and induction method. JA induction resulted in more differentially abundant amplicon sequence variants than SA induction, particularly with Proteobacteria depletion. Conversely, Planctomycetota (bacteria) and Mortierellomycota (fungi) were enriched under both pathways, suggesting that these represent general stress-responsive groups. In the feedback phase, JA- and SA-conditioned soils had no effect on resistance under high aphid pressure, whereas under low aphid density, plants grown in SA-conditioned soil exhibited reduced phloem feeding and lower aphid population development. Together, our results indicate that the benefits provided by the defense-shaped root microbiome depend on pest pressure intensity and arise from overall community shifts rather than specific taxa enrichment. Our findings underscore the complex interactions between plant defense pathways, rhizosphere microbes, and herbivores.

## Introduction

Plants face a myriad of biotic and abiotic stresses and have evolved intricate defense mechanisms, such as stress-specific induced responses, to protect themselves (Kliebenstein 2014). These defense responses are orchestrated by phytohormonal signaling pathways (Pieterse et al. 2012). The induction of specific phytohormonal signaling pathways, and the interplay between them, is what ultimately allows plants to tailor a precise defense response toward a particular abiotic or biotic stress (Aerts et al. 2021, Gilroy and Breen 2022). A prime example of specific induced defenses is the differential activation of the jasmonic acid (JA) and salicylic acid (SA) pathways in response to damage by insect herbivores of distinct feeding guilds or pathogens with different lifestyles. Chewing–biting herbivores, such as caterpillars, or necrotrophic pathogens typically induce the JA pathway, while sap feeders, such as aphids, or biotrophic pathogens, primarily induce the SA pathway (Erb et al. 2012, Ali et al. 2024). The negative interaction between these two pathways (“antagonistic crosstalk”) enables plants to strategically prioritize and fine-tune their defenses according to the type of insect herbivore or attacker encountered (Thaler et al. 2012, Vos et al. 2015).

Besides direct defenses against aboveground herbivores, the induction of JA and SA signaling pathways elicits systemic effects that extend to other plant parts, including the roots. Increasing evidence shows that aboveground induction of plant defenses fundamentally alters the composition and assembly of the microbial communities associated with plant roots (Dastogeer et al. 2020, Eichmann et al. 2021, Friman et al. 2021b). Among these rhizosphere communities, plant-growth-promoting rhizobacteria (PGPR) play a key role in enhancing plant growth and health by facilitating nutrients from the soil and by inducing systemic resistance (ISR) to pathogens and insect herbivores (Pieterse et al. 2014, Rashid and Chung 2017). In addition, JA and SA signaling also influences root morphology and excretion of root exudates, two driving factors that control microbiome assembly belowground (Rolfe et al. 2019, Eichmann et al. 2021, Bagautdinova et al. 2022). For example, aboveground exogenous application of methyl jasmonate (MeJA), the methyl ester of JA used to experimentally induce JA signaling, consistently enriched PGPR taxa in root microbiomes of both monocot (Liu et al. 2017) and dicot plants (Carvalhais et al. 2013). Similarly, aboveground application of SA was shown to increase overall bacterial abundance in roots (Kong et al. 2021), with specific enrichment of beneficial microbes such as *Bacillus subtilis* and other PGPR Gram-positive bacteria following aphid herbivory (Lee et al. 2012, Kim et al. 2015). Notably, disruption of either the JA (Carvalhais et al. 2015) or SA pathways (Lebeis et al. 2015) resulted in altered root microbial communities, confirming their critical role in root microbiome assembly. While these findings highlight a clear connection between induction of defense signaling and microbiome assembly in the rhizosphere, direct evidence linking these changes to microbe-mediated effects on plant resistance is limited.

Plant–soil feedback (PSF) experiments provide a powerful framework to test how these defense-induced microbiome shifts affect plant resistance, as soil-mediated changes are especially driven by changes in soil microbiota (Hu et al. 2018, Hannula et al. 2021). PSF experiments consist of a conditioning phase, in which plants modify the soil microbiome after being challenged with a biotic agent, and a feedback phase, where the effects of these soil-mediated changes are evaluated in a subsequent generation of plants (Kaplan et al. 2018, Mariotte et al. 2018). Using this PSF approach, a few notable studies have shown that defense-induced shifts in root microbiomes can confer resistance against subsequent herbivory or pathogen attack (Berendsen et al. 2018, Hu et al. 2018, Friman et al. 2021a). Nevertheless, the specific role of JA and SA defense pathways in shaping these microbial shifts, and their consequent effects on plant stress resilience, remain poorly understood. For instance, direct comparisons of JA and SA signaling induction effects are scarce (Berendsen et al. 2018, Friman et al. 2021a, Shi et al. 2024), leaving it unclear whether root microbiome changes are pathway-specific. Determining whether and how microbiome assembly differs with the type of defense pathway induced is essential for understanding how plants fine-tune their belowground interactions and how these changes influence plant growth and resistance.

Here, we studied whether aboveground induction of the JA and SA defense signaling pathways in *Brassica oleracea* leads to specific changes in the root microbiome, and whether these defense-induced microbiome shifts influence plant resistance in a subsequent generation of plants. We induced the JA and SA pathways during the conditioning phase of two complementary PSF experiments using two induction methods: (i) foliar application of MeJA or SA solutions and (ii) herbivory by chewing (caterpillars) or phloem-feeding (aphids) insects. Soil conditioned by plants in which the JA or SA defense signaling pathways were induced was collected to characterize bacterial and fungal rhizosphere communities by amplicon sequencing. In the feedback phase, we tested how JA- and SA-conditioned soils influenced plant resistance to caterpillars and aphids. We hypothesized that JA and SA pathway induction would lead to distinct root microbiome assembly. Specifically, we predicted that pathway-specific microbiome shifts would differentially affect resistance to herbivores associated with each defense pathway. We further hypothesized that microbiome restructuring would be primarily determined by the activated defense signaling pathway, i.e. JA or SA, and only to a lesser extent by the induction method, i.e. phytohormone application or insect herbivory.

## Results

### JA and SA pathway induction in phytohormone- and herbivore-treated plants

To confirm successful induction of JA and SA defense signaling during the conditioning phase and to assess whether the two induction methods elicited comparable physiological plant responses, we quantified shoot biomass, endogenous phytohormone levels, and glucosinolate profiles in complementary experiments (Fig. 1; Supplementary Fig. S1a). Shoot biomass differed among treatments in both experiments [generalized linear model (GLM), phytohormone-treated: *χ^2^* = 31.4, df = 2, *P* < 0.001; herbivore-infested: *χ^2^* = 30.6, df = 2, *P* < 0.001; Fig. 2a and Supplementary Table S1]. JA-induced plants, either treated with MeJA or infested with caterpillars, showed a 20%–30% reduction in shoot biomass relative to control plants (Control vs JA, both experiments: *P* < 0.001). In contrast, the shoot biomass of SA-induced plants differed between induction methods. Aphid-infested plants had lower biomass than controls, whereas plants treated with SA did not differ from control plants (Control vs SA; herbivore-infested: *P* = 0.001; phytohormone-treated: *P* = 0.90; Fig. 2a).

**Figure 1:**
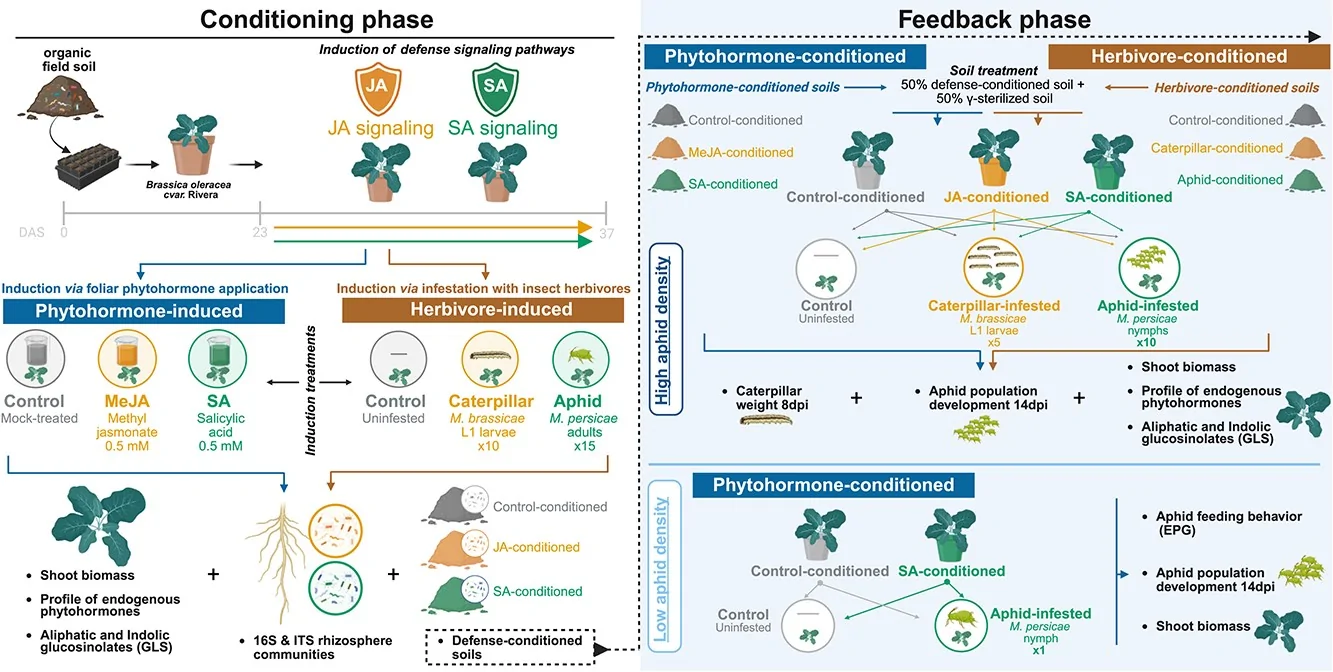
Overview of the PSF experimental design. Two independent experiments, a phytohormone-induced and a herbivore-induced experiment, were performed, each including a conditioning phase followed by a feedback phase. During the conditioning phase, *B. oleracea* plants were grown in organic field soil and the JA or SA defense pathways were induced either by foliar application of phytohormones or by herbivore infestation with caterpillars (*Mamestra brassicae*) or aphids (*Myzus persicae*). Control plants were mock-treated or left uninfested. At harvest, plant traits and rhizosphere microbial communities were characterized, and soils belonging to the same induction treatment were pooled to generate defense-conditioned soils. In the feedback phase, a second generation of plants was grown in a 1:1 mixture of defense-conditioned soil with γ-sterilized soil and challenged with caterpillar or aphid herbivory. Plant performance, insect performance, and aphid feeding behavior were measured using the EPG technique, and plant phytohormone and GLS profiles were measured to assess the consequences of defense-induced soil microbiomes on plant resistance. Figure created in https://BioRender.com.

**Figure 2:**
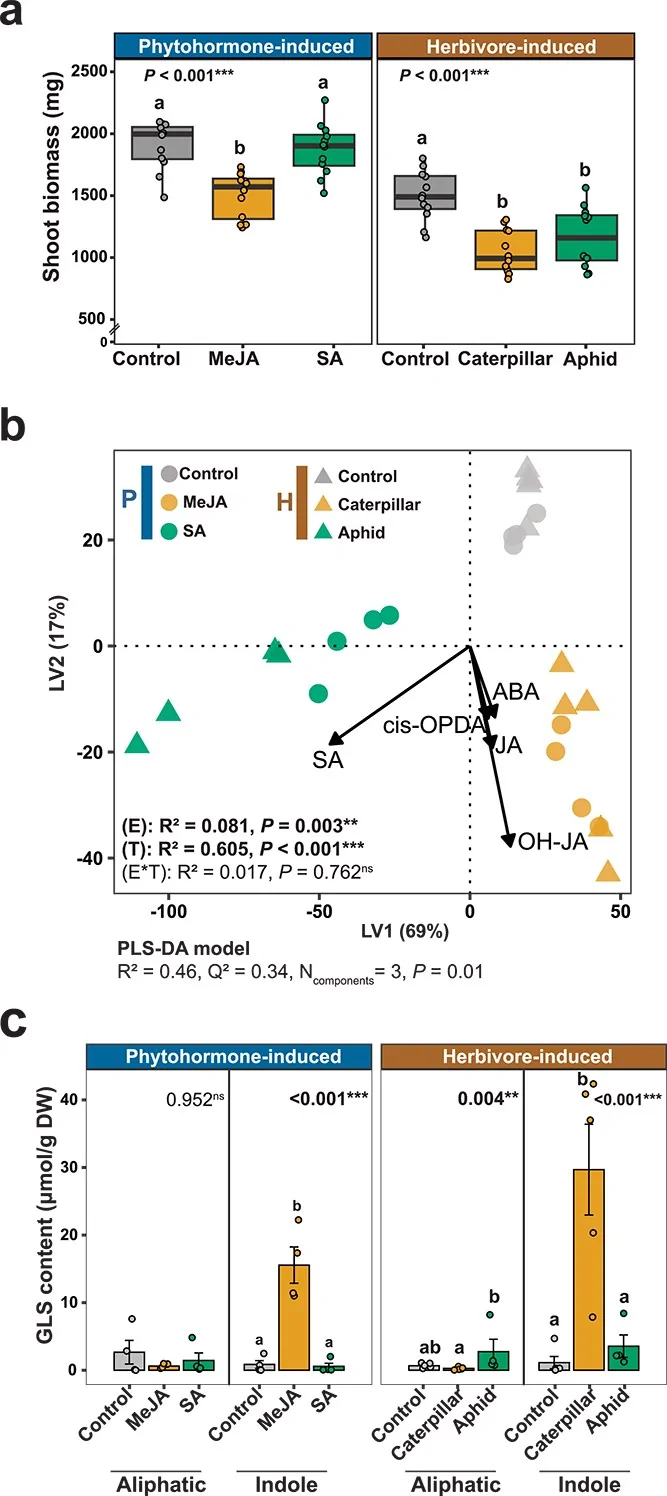
*Brassica oleracea* shoot responses to JA and SA pathway induction 14 days after foliar treatment with phytohormones or herbivore infestation. (a) Shoot biomass; boxes represent the interquartile range (IQR), center lines depict the median values, and whiskers represent data within 1.5× IQR (*n* = 12). (b) Profile of endogenous phytohormones measured in the shoots of both phytohormone-induced (P) and herbivore-induced (H) experiments shown in a partial least squares discriminant analysis (PLSDA) biplot with both scores and loadings. “LV” stands for “latent variable.” Each data point represents a sample. The final PLS-DA model was generated by excluding phytohormones with a VIP score < 0.75. PERMANOVA results are displayed in the plot with experiment (E), induction treatment (T), and their interaction (E^*^T) as factors. (c) GLS concentration in shoots; bar plots show means ± standard error (*n* = 4–5). Circles represent raw data of individual plants. Biomass and GLS data were analyzed with a GLM. ^ns^*P* > 0.05, ^**^P ≤ 0.01, ^***^P ≤ 0.001. Different letters indicate significant differences based on pairwise comparisons at *P* < 0.05.

Although SA application had no effect on plant biomass, multivariate analysis of endogenous phytohormone profiles further confirmed pathway induction. The partial least squares discriminant analysis (PLS-DA) model separated SA-induced plants, regardless of induction method, from both controls and JA-induced plants (*R*^2^ = 0.46, *Q*^2^ = 0.34, *P* = 0.01; Fig. 2b). This separation was mainly associated with high levels of endogenous SA [variable importance in projection (VIP) = 1.78; Supplementary Fig. S2]. Under both induction methods, JA-induced plants were characterized by higher levels of OH-JA (VIP = 1.44), cis-(+)-12-oxo-phytodienoic acid (*cis*-OPDA; VIP = 1.13), and JA (VIP = 0.75; Fig. 2b and Supplementary Fig. S2). Permutational multivariate analysis of variance (PERMANOVA) indicated that induction treatment (control, JA, or SA induction) explained 60% of the variation in phytohormonal profiles compared to only 8% explained by the induction method (Supplementary Table S2).

As glucosinolates (GLS) are key defensive metabolites in brassicaceous plants that contribute to resistance against herbivores and pathogens (Halkier and Gershenzon 2006), their concentrations were quantified to assess downstream defense responses. Both indole and aliphatic GLS subclasses were measured to capture potential pathway-specific changes in defense chemistry. As expected, indole GLS concentrations were higher in both MeJA-treated and caterpillar-infested plants compared to controls (GLM, phytohormone-treated: *χ^2^* = 24.8, df = 2, *P* < 0.001; herbivore-infested: *χ^2^* = 37.5, df = 2, *P* < 0.001), whereas aliphatic GLS concentrations did not differ among induction treatments under either induction method (Fig. 2c and Supplementary Table S1).

### Bacterial and fungal communities show specific and general responses to JA and SA pathway induction

To characterize the root microbiome response to aboveground JA- and SA pathway induction, we analyzed bacterial and fungal rhizosphere communities by 16S rRNA gene and internal transcribed spacer (ITS) amplicon sequencing in plants in which these pathways were induced via foliar phytohormone application or herbivore infestation in the conditioning phase. Overall, PERMANOVA analyses revealed that bacterial community composition was more strongly influenced by the induction method (*R*^2^ = 0.13, *F* = 13.37, df = 1, *P* < 0.001; Fig. 3a) than the fungal community (*R*^2^ = 0.04, *F* = 3.47, df = 1, *P* < 0.001; Fig. 4a). When microbial communities were analyzed independently by the type of induction method, induction treatment had an effect on the bacterial composition only in herbivore-infested plants (Treatment: *R*^2^ = 0.06, *F* = 1.32, df = 2, *P* = 0.022; Fig. 3b), whereas fungal composition was affected only in phytohormone-treated plants (Treatment: *R*^2^ = 0.12, *F* = 1.94, df = 2, *P* < 0.001; Fig. 4b). Alpha-diversity metrics [richness, Shannon index, and Faith’s phylogenetic diversity (PD)] showed no differences between treatments for bacterial communities under either induction method (Supplementary Fig. S3). For fungal communities, the effect on diversity varied with the induction method: a decrease in species richness and Faith’s PD was observed only under phytohormone application in MeJA-treated plants (Control vs JA: richness: *P* = 0.002, Faith’s PD: *P* = 0.015; Supplementary Fig. S4). Together, these results indicate that both the defense signaling pathway (JA or SA) and induction method (phytohormone application or insect herbivory) influenced the rhizosphere microbial composition, and that bacterial and fungal communities responded differently to these factors.

**Figure 3:**
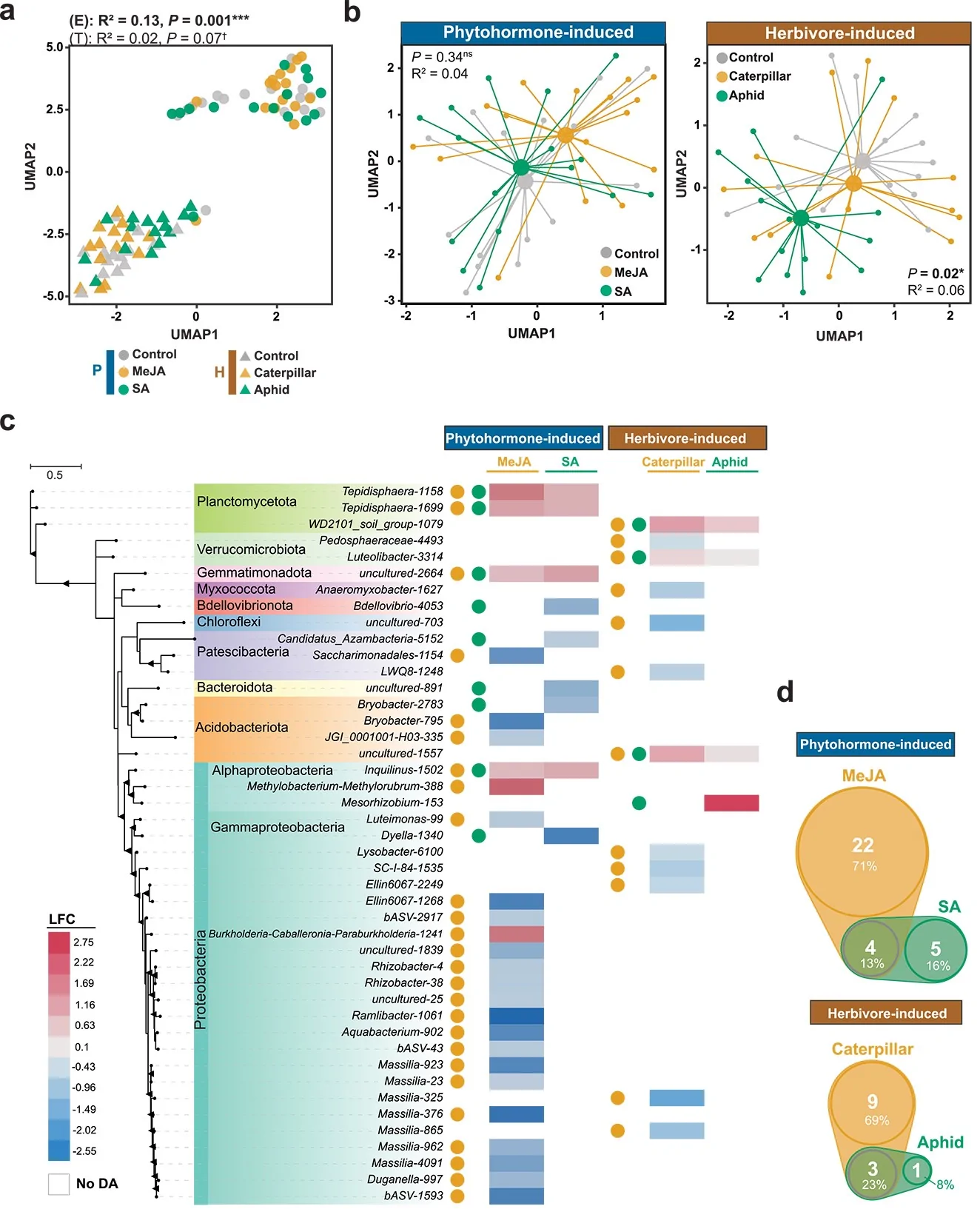
Changes in root bacterial communities after aboveground induction of the JA or SA pathway. (a) UMAP plot of UniFrac distances showing bacterial (16S) community composition across both phytohormone-induced (P) and herbivore-induced (H) experiments. PERMANOVA results are displayed in the plots with experiment (E) and induction treatment (T) as factors. (b) UMAP plots of bacterial communities for each experiment analyzed independently. Each data point represents individual rhizosphere samples from one plant; big circles represent centroids. PERMANOVA results are displayed in the plots with induction treatment as a factor. (c) Inferred maximum-likelihood phylogenetic tree of 44 DA ASVs. Branch lengths represent the number of nucleotide substitutions per site and triangles indicate branches with bootstrap support ≥ 90%. ASVs are annotated by phylum, shown in boxes next to the tree, and genus (when available) followed by unique ASV identifiers. Circles in the treatment columns indicate DA ASVs relative to the corresponding controls; within each experiment, the first and second columns represent JA and SA induction, respectively. The heatmap shows LFC in abundance relative to controls, with positive and negative values indicating increased and decreased abundance, respectively. Blank spaces represent nonsignificant differences in abundance between stress-induced treatments and controls. (d) Venn diagrams summarizing the total number of unique and shared DA ASVs between JA- and SA-induced plants versus controls for both experiments.

**Figure 4:**
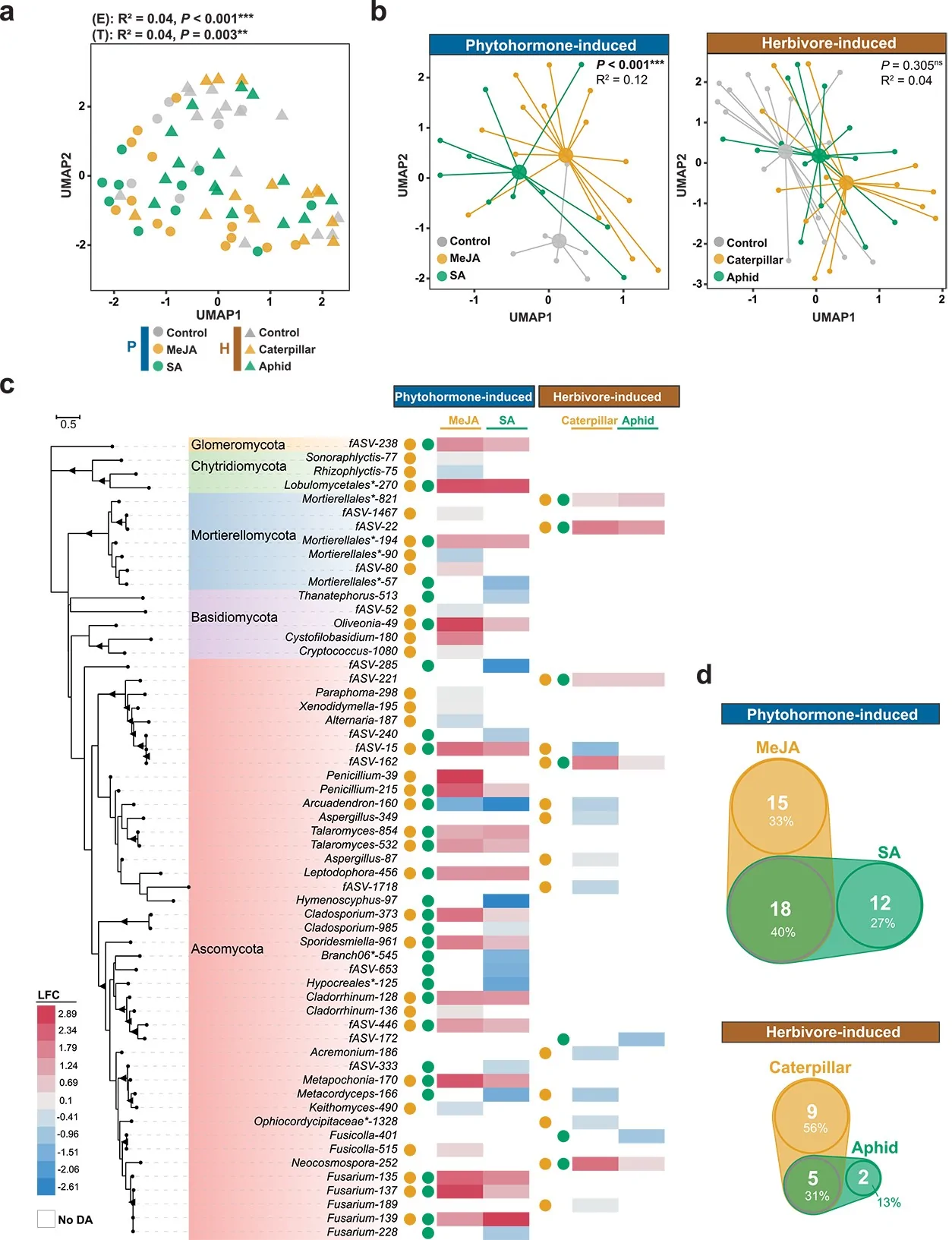
Changes in fungal communities after JA and SA pathway induction. (a) UMAP plot of UNIFRAC distances showing fungal (ITS) community composition, across both phytohormone-induced (P) and herbivore-induced (H) experiments. PERMANOVA results are displayed in the plots with experiment (E), and induction treatment (T) as factors. (b) UMAP plots of fungal communities for each experiment analyzed independently. Each data point represents individual rhizosphere samples from one plant; big circles represent centroids. PERMANOVA results are displayed in the plots with induction treatment as a factor. (c) Inferred maximum-likelihood phylogenetic tree of 58 DA ASVs. Branch lengths represent the number of nucleotide substitutions per site and triangles indicate branches with bootstrap support ≥ 90%. ASVs are annotated by phylum, shown in boxed next to the tree, and genus (when available) followed by unique ASV identifiers. The (^*^) in the ASV name stands for “genus *incertae sedis*”. Circles in the treatment columns indicate DA ASVs relative to the corresponding controls; within each experiment the first and second columns represent JA and SA induction, respectively. The heatmap shows LFC in abundance relative to controls, with positive and negative values indicating increased and decreased abundance, respectively. Blank spaces represent nonsignificant differences in abundance between stress-induced treatments and controls. (d) Venn diagrams summarizing the total number of unique and shared DA ASVs between JA- and SA-induced plants and controls for both the experiments.

Differential abundance analyses using Analysis of Compositions of Microbiomes with Bias Correction (ANCOM-BC) revealed that phytohormone-treated plants harbored at least twice as many differentially abundant (DA) amplicon sequence variants (ASVs) as herbivore-infested plants for both bacteria and fungi (Figs. 3d and 4d). For bacteria, ~70% of the DA ASVs were specifically associated with JA pathway induction rather than SA induction (Fig. 3d). Notably, most of the JA-responsive ASVs were less abundant in both MeJA-treated and caterpillar-infested plants (Fig. 3c). The majority of the JA-responsive ASVs were Proteobacteria, with the genus *Massilia* particularly affected, showing a decrease in abundance of 7 ASVs out of 44 total DA ASVs (Fig. 3c). In contrast, SA-responsive ASVs were fewer and less pathway-specific. Aphid-infested plants had only four DA ASVs, three of which overlapped with caterpillar-infested plants and one involved a unique enrichment of a *Mesorhizobium* ASV (Fig. 3d). Similarly, in SA-treated plants, about half of the DA ASVs were also responsive to MeJA treatment. These JA and SA dual-responsive ASVs were more abundant under induction treatments compared to controls, suggesting a broad enrichment of stress-associated microbes regardless of the defense pathway induced. Specifically, Planctomycetota bacteria were predominantly enriched in both experiments under JA and SA induction (Fig. 3c).

For fungi, the proportion and distribution of DA ASVs differed between induction methods. In phytohormone-treated plants, MeJA and SA treatments yielded similar numbers of DA ASVs (~50% each), whereas caterpillar-infested plants had twice as many DA ASVs as aphid-infested plants (Fig. 4d), mirroring bacterial patterns. Most fungal DA ASVs were enriched in JA- and SA-induced plants compared to controls, with the majority belonging to the phylum Ascomycota (Fig. 4c). Fungal responses were less pathway-specific than bacterial responses, with 30%–40% of all DA ASVs overlapping between JA and SA induction under both induction methods (Fig. 4d). ASVs responsive to both JA and SA were found within the phylum Mortierellomycota across both phytohormone-treated and herbivore-infested plants, as well as in the *Fusarium* and *Neocosmospora* genera, both belonging to the Nectriaceae family (Fig. 4c).

In summary, JA and SA pathway induction significantly altered the rhizosphere microbial composition and enrichment, with effects depending on the induction method. Phytohormone treatments had a stronger impact than herbivore infestations, for both bacterial and fungal communities. Bacteria responded more specifically to JA induction with a decreased abundance of several ASVs, whereas fungi showed greater overlap between the two defense signaling pathways. Notably, conserved groups of JA and SA dual-responsive microbes, represented by specific taxa in both bacteria and fungi, were consistently enriched in response to both pathways. These findings highlight the intricate interplay between aboveground defense signaling and rhizosphere microbial dynamics.

### JA- and SA-conditioned soils show limited effects on plant resistance under high herbivore pressure

During the feedback phase, we assessed the bottom-up effect of microbiome changes associated with JA and SA signaling. A second generation of *B. oleracea* plants was grown in soil conditioned by plants in which either the JA or the SA pathway had been induced, via phytohormone application (MeJA or SA), or by herbivore infestation (caterpillars or aphids). These plants were subsequently subjected to herbivory by *M. brassicae* caterpillars or *M. persicae* aphids to evaluate soil-mediated effects on plant resistance to insects from different feeding guilds (Fig. 1; Supplementary Fig. S1b).

There was no effect of soil treatment or herbivory on plant biomass (GLM soil treatment: phytohormone-conditioned: *χ^2^* = 3.48, df = 2, *P* = 0.18; herbivore-conditioned: *χ^2^* = 2.34, df = 2, *P* = 0.31; GLM herbivory: phytohormone-conditioned: *χ^2^* = 3.48, df = 2, *P* = 0.45; herbivore-conditioned: *χ^2^* = 5.68, df = 2, *P* = 0.058; Fig. 5a and Supplementary Table S3). The PLS-DA model of endogenous phytohormone profiles showed a strong clustering according to herbivore identity (caterpillars or aphids) during the feedback phase, irrespective of soil treatment and the induction method used during the conditioning phase (PLS-DA, *R*^2^*X* = 0.88, *R*^2^*Y* = 0.39, *Q*^2^ = 0.37, *P* = 0.01; Fig. 5b). Consistently, PERMANOVA analysis indicated that herbivory explained 35% of the variation in endogenous phytohormone profiles, compared to only 5% explained by the induction method in the conditioning phase and 2% by soil treatment (Supplementary Table S2), indicating that herbivory was the primary driver of plant responses in the feedback phase. Caterpillar feeding was associated with higher levels of JA-related phytohormones (*cis*-OPDA, OH-JA, and JA), whereas aphid feeding was associated with increased SA levels (Fig. 5b).

**Figure 5:**
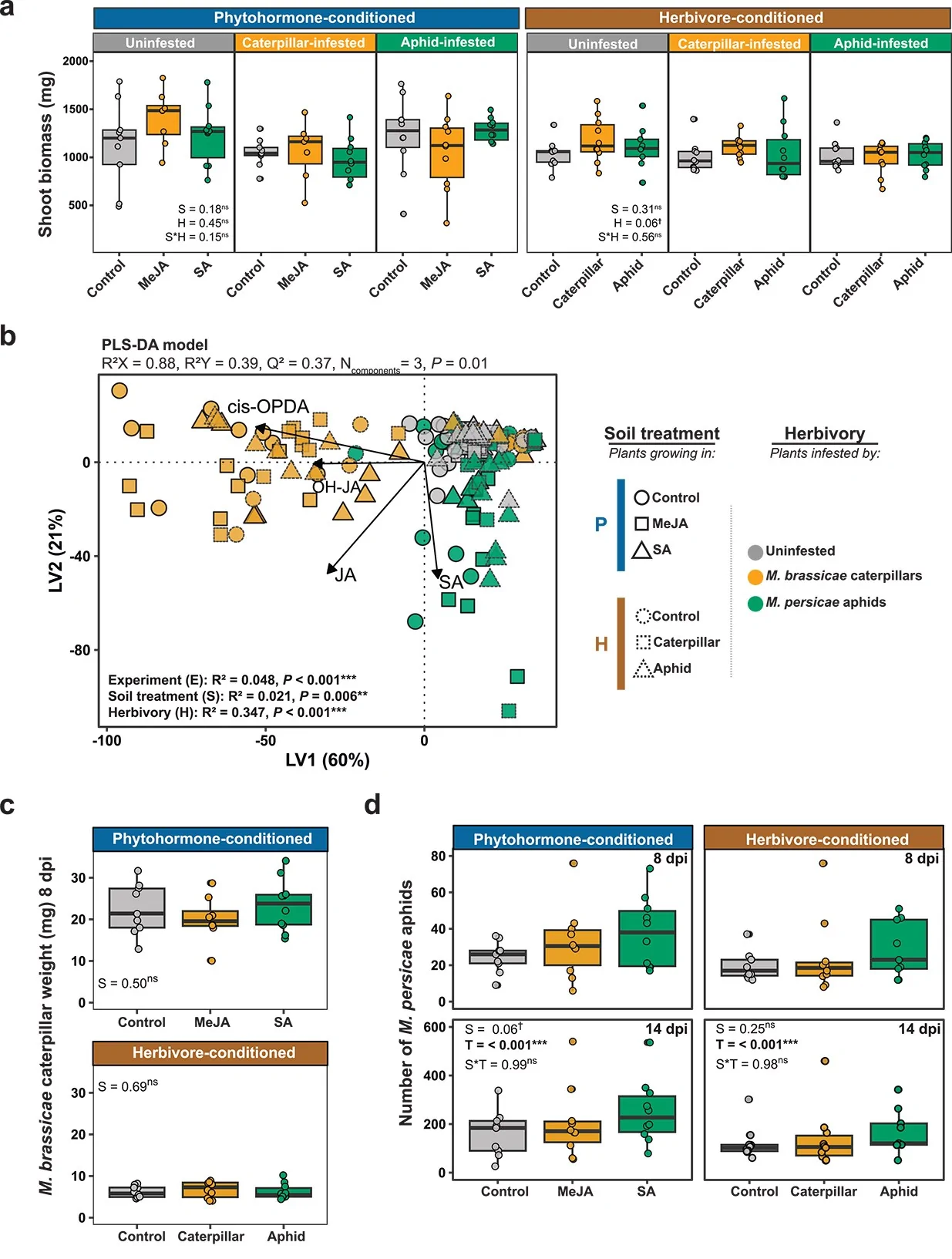
Plant traits and herbivore responses to defense-induced soil treatments under high herbivore pressure in the feedback phase. Second-generation plants were challenged with 5 L_1_ caterpillar or 10 neonate aphids to evaluate their resistance to herbivory in the feedback phase. (a) Dry shoot biomass (*n* = 10). (b) PLS-DA biplot of endogenous phytohormone profiles in the shoots. Data points represent individual plant samples (biological replicates) and are colored by herbivory type during the feedback phase. Shapes indicate soil treatment type, and outlines distinguish phytohormone-conditioned (full line) from herbivore-conditioned (dashed line) experiments. “LV” stands for “Latent Variable.” The final model was generated by excluding phytohormones with VIP scores < 0.75. PERMANOVA results are displayed in the plot. (c) Average weight of *M. brassicae* caterpillars per plant, measured 8 dpi (*n* = 9–10). (d) Population development of *M. persicae* aphids at 8 and 14 dpi following initial infestation with 10 neonate nymphs per plant (*n* = 9–10). Boxplot boxes represent the IQR, center lines indicate medians, and whiskers show 1.5× IQR. Circles represent raw data of individual plants. Biomass and herbivore performance data were analyzed with a GLM using soil treatment (S), herbivory type (H), and time (T) as the main factors, including their interactions where applicable. ^ns^*P* > 0.10, †*P* ≤ 0.10, ^*^P ≤ 0.05, ^**^P ≤ 0.01, ^***^P ≤ 0.001.

GLS levels showed a similar pattern to that of phytohormones, with herbivory having a stronger effect than soil treatment (Supplementary Fig. S5 and Supplementary Table S3). In both experiments, herbivory affected concentrations of both aliphatic and indolic GLS (GLM aliphatic GLS: phytohormone-conditioned: *χ^2^* = 110.25, df = 2, *P* < 0.001; herbivore-conditioned: *χ^2^* = 26.29, df = 2, *P* < 0.001; GLM indole GLS: phytohormone-conditioned: *χ^2^* = 127.51, df = 2, *P* < 0.001; herbivore-conditioned: *χ^2^* = 56.56, df = 2, *P* < 0.001). Specifically, caterpillar feeding increased indole GLS and reduced aliphatic GLS compared to uninfested controls (uninfested vs caterpillar-infested, both experiments: *P* < 0.001; Supplementary Fig. S5).

To further evaluate the effects of soil treatment on plant resistance, we measured the performance of *M. brassicae* caterpillars and *M. persicae* aphids feeding on plants grown in JA- or SA-conditioned soils. Notably, caterpillars were overall ~3.5 times heavier on plants growing in phytohormone-conditioned soils compared to herbivore-conditioned soils, regardless of pathway identity (Fig. 5c). Despite differences in absolute caterpillar weight between experiments, soil treatment did not affect caterpillar performance at 8 days postinfestation (dpi) in either case (GLM, phytohormone-conditioned: *χ^2^* = 1.37, df = 2, *P* = 0.50; herbivore-conditioned: *χ^2^* = 0.73, df = 2, *P* = 0.69; Fig. 5c and Supplementary Table S3). A similar pattern was observed for aphids; the total number of aphids at 14 dpi was overall ~1.5 times higher in the phytohormone-conditioned experiment (*x-* = 206.62 aphids) compared to the herbivore-conditioned experiment (*x-* = 142.03 aphids; Fig. 5d). Although not significant, a trend emerged in the phytohormone-conditioned experiment, suggesting higher reproduction of *M. persicae* aphids on plants grown in SA-soil compared to controls at both 8 and 14 dpi (GLM, phytohormone-conditioned: *χ*^2^ = 5.68, df = 2, *P* = 0.058; Fig. 5d and Supplementary Table S3). Overall, our results from the feedback phase indicate that the effect of soil treatment on plant growth and resistance was limited, with only a mild influence on aphid performance.

### Low aphid pressure reveals positive plant–soil feedback on plant resistance

To explore the hypothesis that the high aphid herbivory pressure in the previous experiment (starting with 10 aphids per plant and building up to hundreds of aphids) masked the subtle effects of defense-induced soil microbiomes, we conducted an additional PSF experiment. In this follow-up experiment, we used a lower initial aphid density of a single *M. persicae* aphid per plant. A second generation of plants was grown on soils conditioned by SA-treated or mock-treated control plants. After introducing one adult aphid per plant, we measured feeding behavior using the electrical penetration graph (EPG) technique (Tjallingii 1988). Thereafter, one neonate nymph was left per plant and population development, as well as plant biomass, was recorded at 14 dpi (Fig. 1; Supplementary Fig. S6).

Our results showed that aphids spent overall more time in the pathway phase (i.e. not feeding) on plants grown in SA-conditioned soil compared to those grown in control-conditioned soil (Mann–Whitney *U* test, *P* = 0.012; Fig. 6a). While the total phloem feeding time did not differ between soil treatments (Supplementary Table S4), time-trend analyses indicated reduced phloem feeding activity in SA-conditioned plants during the later half of the 8-h recording (generalized linear mixed-effects model, GLMM, Soil treatment^*^Time: *χ^2^* = 135.54, df = 7, *P* < 0.001; Fig. 6b and Supplementary Table S5). Other behavioral variables, such as salivation in the phloem and xylem ingestion, were unaffected (Supplementary Table S4). Interestingly, the subtle effects on feeding behavior of adult aphids were reflected in the performance of their offspring. Aphids feeding on plants growing on SA-conditioned soil exhibited 30% lower reproduction compared to those on control-conditioned soil (GLM, *χ^2^* = 8.80, df = 1, *P* = 0.003; Fig. 6c and Supplementary Table S5). These differences in aphid performance were independent of plant biomass, which remained unchanged between soil treatments (GLM, *χ^2^* = 1.76, df = 1, *P* = 0.184; Fig. 6d and Supplementary Table S5).

**Figure 6:**
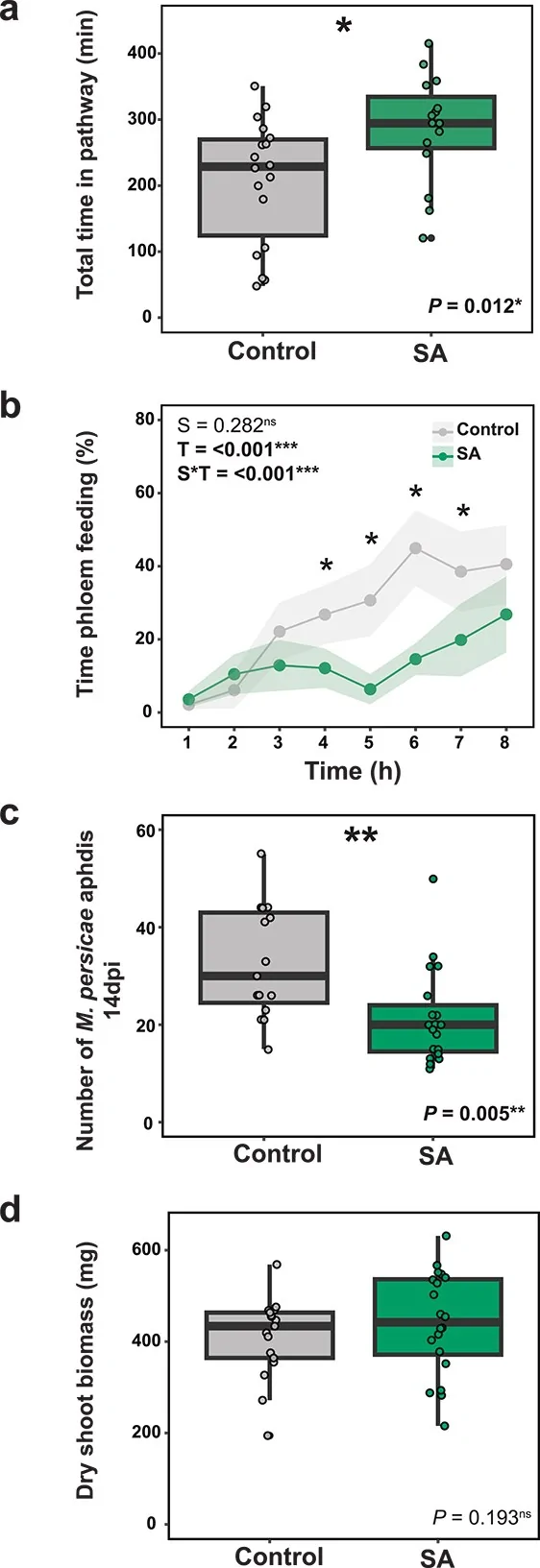
Plant and aphid responses to SA-conditioned soil starting from low aphid density. (a) Total time spent in the pathway phase by *M. persicae* aphids on plants grown in control and SA-conditioned soils during the 8-h EPG recording (*n* = 15–18). (b) Percentage of time spent feeding on the phloem by *M. persicae* aphids during the same recording. Dots represent mean values and shaded areas indicate standard error. (c) Aphid population development measured 14 dpi, following the initial infestation of a single nymph per plant (*n* = 15–19). (d) Dry shoot biomass measured at harvest (*n* = 17–20). Boxplot boxes represent the IQR, center lines indicate medians, and whiskers show 1.5× IQR. Circles represent raw data of individual plants. Total time in pathway was analyzed with a Mann–Whitney *U* pairwise comparison test. Time-trend analyses for time phloem feeding were conducted with a GLMM with soil treatment (S) and time (T) as the main factors, including their interaction, and plant ID as a random factor. Plant biomass and aphid number were analyzed with a GLM. ^ns^*P* > 0.05, ^*^P ≤ 0.05, ^**^P ≤ 0.01, ^***^P ≤ 0.001.

## Discussion

### Insect herbivory drives microbiome assembly in a unique way

Our study explored the role of induced plant defenses via the JA and SA pathways in shaping rhizosphere microbial communities and their subsequent effects on plant resistance to herbivores from different feeding guilds. Using PSF experiments, we tested whether microbiome shifts induced by JA and SA defense signaling were pathway-specific and beneficial for plant resistance. We found that induction of the JA and SA pathways, through either foliar application of phytohormones or insect herbivory, led to both pathway-specific and general shifts in the rhizosphere microbiome. The method of induction (phytohormone application vs insect herbivory) strongly influenced these shifts, with more DA ASVs responding to phytohormone application than to insect herbivory. Phytohormone treatments generally downregulated bacterial ASVs and enriched fungal ASVs, regardless of the pathway. The limited overlap in DA ASVs between phytohormone- and insect-mediated induction, with the exception of *Massilia* suppression by both MeJA and caterpillars, suggests that insect feeding imposes a unique microbiome assembly distinct from inducing defense signaling with elicitors such as MeJA and SA applications. In our study, soils exhibited a pest-suppressive effect only under specific conditions—when plants where grown in SA-conditioned soil and exposed to low aphid densities—indicating a context-dependent role of these microbiomes in plant defenses.

### Exogenous phytohormones have a stronger impact on the microbiome

Phytohormone treatments resulted in approximately twice as many DA ASVs as herbivore infestations, suggesting a stronger impact on microbial community composition. Since both treatment types elicited similar effects on plant biomass, endogenous phytohormone levels, and GLS profiles in the conditioning phase, this difference is unlikely due to differences in the magnitude of the pathway induction. It is important to acknowledge that due to space limitations, experiments were conducted sequentially rather than simultaneously, which may have introduced subtle environmental variation such as fluctuations in greenhouse temperature, humidity, or day length. Notwithstanding, the soil used for both came from the same batch, therefore providing a consistent microbial background between experiments. Hence, these observed differences may reflect intrinsic variation in the signaling networks and physiological responses activated during herbivory compared to those triggered by exogenous phytohormone application. For instance, wounding, MeJA application, and caterpillar feeding all activate overlapping JA and SA transcriptional responses; however, the magnitude, timing, and specific genes involved differed substantially between treatments, and even among different caterpillar species (Vogel et al. 2007). Likewise, MeJA and caterpillar damage elicit similar, but not identical, metabolic responses in leaves, with MeJA in some cases triggering stronger accumulation of certain GLS and other defense-related metabolites (Papazian et al. 2019). Therefore, one possible explanation is that these signaling and metabolic differences extend belowground, where phytohormone- and herbivory-specific responses could alter carbon sources and the defense compounds exuded by the roots, thereby influencing rhizosphere microbial assembly. This hypothesis remains to be tested, as root-exudate profiles were not measured in the present study. Future studies testing how the root-exudate composition varies under different modes of defense-signaling induction would provide critical insights into the mechanisms driving these microbiome differences.

### JA-mediated decline in *Massilia*

Induction of the JA pathway had a stronger effect on the bacterial rhizosphere microbiome than induction of the SA pathway, with a higher number of differentially abundant ASVs in both MeJA and caterpillar treatments. This aligns with previous studies in *Arabidopsis* where the JA pathway was strongly associated with changes in root exudates and root microbiome composition (Carvalhais et al. 2013, 2015, 2017, Kulkarni et al. 2024). In contrast, the induction of the SA pathway appears to have more subtle or context-dependent effects. For example, moderate suppression of SA-regulated defenses has been linked with an increase in microbial colonization (Zamioudis and Pieterse 2012), suggesting that SA induction may inhibit the proliferation or recruitment of certain microbial taxa, as observed in this study. In fact, a similar pattern was observed in *Sedum alfredii*, where a study by Shi et al. (2024) reported that JA-treated plants had a higher number of responsive rhizosphere bacterial and fungal operational taxonomic units (OTUs) compared to SA-treated plants. Remarkably, most JA-responsive bacterial ASVs in our study were depleted on the roots relative to controls, such as members of the genus *Massilia*, indicating that plants might actively exclude specific bacteria through JA-induced defenses. Similar results have shown that the *Massilia* abundance halved after MeJA treatment of soil (Carvalhais et al. 2014), and decreased in the rhizosphere of tomato plants exposed to caterpillar herbivory (Malacrinò and Bennett 2024). While *Massilia* species possess potential PGPR traits such as root colonization, abiotic stress tolerance, phosphorus solubilization, and production of indole-3-acetic acid (Kuffner et al. 2010, Ofek et al. 2012, Li et al. 2024a), their susceptibility to competition with other rhizosphere bacteria (Ofek et al. 2012), which were enriched after JA induction, may explain their decline.

### Shared and unique microbes upon JA- and SA-pathway induction

We observed enrichment of specific bacterial and fungal ASVs by both the JA and SA pathways across both experiments. These findings suggest that there is a group of general-stress-responsive microbes that are enriched when plants are exposed to biotic attack, regardless of the feeding guild or defense pathway induced. Other studies have also identified shared microbial responses to JA and SA pathways. For instance, ASVs from the *Rhodanobacter* and *Nocardia* genera were enriched after both caterpillar and aphid herbivory (Friman et al., 2021a), while ASVs from *Sphingomonas* and *Burkholderia*, *Paraburkholderia*, and *Caballeronia* groups were enriched in *S. alfredii* plants treated with JA and SA (Shi et al. 2024). In our study, some of these general stress-responsive microbes appeared to be conserved within specific taxonomical groups, particularly the Planctomycetota for bacteria and the Mortierellomycota for fungi. Fungi belonging to the Mortierellomycota have been previously described as being plant-growth-promoting (Ozimek and Hanaka 2020), and have been found in higher relative abundance in healthy roots of *Brassica rapa* plants compared to those diseased with bacterial soft rot (Li et al. 2024b). Similarly, Planctomycetota bacteria, which are ubiquitous in soil environments and known for their secondary metabolite biosynthesis (Buckley et al. 2006, Kallscheuer and Jogler 2021), have been associated with antimicrobial resistance (Godinho et al. 2024). Given that both the JA and SA pathways induce antimicrobial gene activity in plants (Thomma et al. 1998), it is possible that plants in which defense pathways are induced select for antimicrobial resistance in their microbiome. While these conserved taxonomic patterns across JA- and SA-induced plants suggest that defense signaling may favor specific microbial groups that are potentially beneficial, taxonomic shifts alone cannot resolve their functional outcomes (Louca et al. 2016). Future studies integrating shotgun metagenomic approaches and experimental functional validation would help determine whether defense-enriched microbial groups contribute with specific traits related to plant growth, pathogen suppression, or stress resilience.

### Aphid density modulates positive SA-driven plant–soil feedback effects

In our study, plants exposed to aphid populations > 200 insects after 2 weeks did not show a significant PSF effect, while plants exposed to final aphid populations < 50 insects did reveal a significant impact of soil treatment. This is in line with other reports of microbe-induced resistance against aphids, where aphid densities were usually at a comparable low level, starting with only two to four nymphs per plant (Rashid et al. 2017, Zytynska et al. 2020, Xi and Zytynska 2022). We found that aphid populations were suppressed on plants growing in SA-conditioned soil compared to the control, consistent with our initial hypothesis. Furthermore, EPG recordings showed that aphids experienced greater difficulty in establishing phloem feeding on plants grown in SA-conditioned soil. The combination of reduced phloem feeding and aphid reproduction is indicative of either increased phloem-located defenses and/or decreased nutrient content of the phloem sap (Kloth et al. 2017, Jakobs et al. 2018). Measuring the phloem content was beyond the scope of this study, but it could help determine in future studies the mechanisms of aphid suppression in SA-conditioned soils.

Our findings suggest that PSF effects on aphids were dependent on the density of the aphid population during the feedback phase. The importance of aphid density in modulating plant defenses is well documented (Kroes et al. 2015, 2017a, 2017b, Ponzio et al. 2017). Low aphid densities (e.g. 5 nymphs) upregulate SA-dependent defense genes, while high aphid densities (e.g. 25 aphids) have been linked to the suppression of these genes and the activation of JA-dependent defenses instead (Kroes et al. 2015). Notably, high aphid densities suppress genes involved in carbohydrate metabolism, photosynthesis, and bacterial responses mediated by WRKY transcription factors (Kroes et al. 2017a). Higher aphid densities could thus have resulted in differential metabolic responses that overrode PSF effects. Whether or not it is a general trend that the defense-induced soil microbiome can only mitigate low aphid densities remains to be investigated.

Overall, our findings emphasize the complex interplay between plant signaling pathways, rhizosphere microbial communities, and insect herbivores and underline the importance of experimental conditions in evaluating the ecological and functional significance of defense-induced microbiomes. Further studies focusing on root-exudate dynamics and microbial functional traits will be essential to unravel the mechanisms underlying these interactions and their implications for sustainable pest management.

## Materials and Methods

### Study system

*Plants.* Plants of white cabbage, *B. oleracea* L. var. *capitata* cv. ‘Rivera’, were used for all the experiments. Seeds of *B. oleracea* were uncoated, with a homogeneous genetic background (F_1_), and provided by Bejo Zaden B.V. (Warmenhuizen, the Netherlands). For germination, seeds were stratified for 4 days at 4°C in a Petri dish with a filter paper and 2.5 mL of ultrapure water (Milli-Q®, Merck). After stratification, seeds were left for 1 day at greenhouse temperature before sowing. All the plants were grown in a greenhouse compartment at 23 ± 2°C, in a 16 h:8 h light:dark photoperiod, and in 50%–60% relative humidity.

*Insects.* The generalist caterpillar *M. brassicae* L. (Lepidoptera: Noctuidae) and the generalist green peach aphid *M. persicae* (Hemiptera: Aphididae) were used as models for chewing–biting and phloem-feeding herbivory, respectively. Both insect species were reared on Brussels sprout plants (*B. oleracea* var. *gemmifera*) under controlled conditions (22 ± 2°C, 70 ± 10% RH, and a 16 h:8 h light:dark photoperiod). To obtain insects of the same age for the infestations, 1-day-old eggs of *M. brassicae* were gently transferred to *B. oleracea* leaves 6 days before experiments, producing synchronized first-instar (L_1_) caterpillars. For *M. persicae*, adults were allowed to lay nymphs on *B. oleracea* plants for 24 h, after which the adults were removed. The resulting nymphs developed for 7 days and the resulting 8-day-old adults were used for the experiments.

*Soil.* The soil used in this study, hereafter called “MiCRop” soil, was obtained from the upper 80-cm layer of an organic agricultural field in Nergena area, Bennekom (the Netherlands, coordinates: N 51.996250 E 5.659375). Since its original collection in 2014, the soil had been kept outdoors unmanaged on a heap near a field location. The MiCRop soil used in this study came from a single batch collected from the heap in April 2021. After collection, the soil was air-dried, passed through a 5-mm sieve, mixed, and stored in 10-kg bags in dark conditions until use. Half of the bagged soil was γ-irradiated (>25 kGy gamma irradiation, Isotron, Ede, the Netherlands) for use as sterile background soil in the feedback phase. The nutrient content and physicochemical properties of the soil were analyzed by Eurofins Agro (Wageningen, the Netherlands; Supplementary Table S6).

### Plant–soil feedback experiments

Using PSF experiments, we investigated how aboveground induction of JA and SA defense pathways affects belowground microbial communities and, in turn, plant and insect performance. Each PSF experiment consisted of two phases: a conditioning phase, in which soil inoculum was generated by inducing defense responses in a first generation of plants, and a feedback phase, in which conditioned soil was used to grow a second generation of plants (Kaplan et al. 2018). Two complementary PSF experiments were conducted: the “phytohormone-treated” experiment, in which defense pathways were induced via exogenous MeJA or SA application, and the “herbivore-infested” experiment, in which induction was made through herbivore infestation with either caterpillars or aphids (Fig. 1; Supplementary Fig. S1).

*Conditioning phase: top-down effects of defense induction.* For the conditioning phase, two stratified seeds of *B. oleracea* were sown in 1 L pots filled with MiCRop soil. After germination, 7 days after sowing (DAS), only one seedling per pot was retained. All the plants were watered and fertilized with ~60 mL of half-strength Hoagland solution (5.6 mM NH_4_NO_3_, 0.4 mM K_2_HPO_4_, 0.8 mM MgSO_4_, 0.8 mM K_2_SO_4_, 1.6 mM CaCl_2_, 0.18 mM FeSO_4_, 0.18 mM Na_2_EDTA, 23 *μ*M H_3_BO_3_, 4.5 *μ*M MnCl_2_, 0.3 *μ*M CuSO_4_, 1.5 *μ*M ZnCl_2_, 0.1 *μ*M Na_2_MoO_4_) weekly until harvest. Stress induction began at 23 DAS and lasted for 14 days in both experiments.

For the phytohormone-treated experiment, the entire aboveground leaf area of plants was dipped in a solution of either 0.5 mM MeJA or 0.5 mM SA containing 0.015% Silwet®L-77 as a surfactant, and 0.1% ethanol, following the protocol of Berendsen et al. (2018). Control plants were mock-treated by dipping them in a solution containing 0.015% Silwet®L-77 and 0.1% ethanol only. Plants were dipped every 3 days for a total of four times to ensure continuous stress induction. For the herbivore-infested experiment, plants were infested on the first fully developed leaf with either 10 L_1_  *M. brassicae* caterpillars (Caterpillar) or 15 8-day-old wingless *M. persicae* adults (Aphid). Control plants were left uninfested. The number of insects per plant was verified 24 h after infestation to ensure consistency. Insects were left to feed on the plants until harvest (Fig. 1; Supplementary Fig. S1a). A completely randomized design was used to assign treatments to plants in both experiments. Each treatment had 16 biological replicates, resulting in a total of 48 plants per conditioning phase.

Plants were harvested 37 DAS, 2 days after the last dipping treatment for the phytohormone-induced experiment. At harvest, six to nine leaf discs of 1 cm diameter were collected from the three youngest fully expanded leaves of five randomly selected plants, placed in 1.5 mL tubes, and flash frozen in liquid nitrogen. Insect herbivores were removed from plants before taking the samples for the herbivore-infested experiment. Samples were stored at −80°C until processing for measuring endogenous phytohormone and GLS concentrations. Once leaf-disc samples were taken, shoots from all the plants were cut at soil level. Subsequently, the complete root systems were carefully removed by hand, with loosely attached soil gently shaken off, leaving a thin layer of soil around the roots, which was considered the rhizosphere. Shoots were dried for 3 days at 70°C to measure the dry biomass, and roots were stored in 15 mL tubes at −20°C until processing for DNA extraction and microbiome community profiling. The conditioned soil consisted of all the bulk soil, including the loosely attached soil shaken off the roots, collected from the 16 pots within each specific treatment. Since the roots had fully occupied the pots by the end of the conditioning phase, we considered the entire soil volume to be influenced by root activity. The conditioned soil was then mixed thoroughly and air-dried at room temperature for 5 days to prevent the growth of opportunistic fungi under humid conditions. Finally, conditioned soils were stored at 4°C until their use in the feedback phase. The phytohormone-induced conditioning phase started on 12 May 2021, and the herbivore-induced conditioning phase started 6 weeks later on 22 June 2021.

*Feedback phase: bottom-up consequences on plant performance.* In the feedback phase, we tested the effects of defense-conditioned soils on plant growth and resistance to insect herbivory. Defense-conditioned soils originated from plants in which defense pathways had been induced either by phytohormone application (MeJA, SA, or mock control) or by herbivore infestation (caterpillars, aphids, or uninfested control) during the conditioning phase (Fig. 1; Supplementary Fig. S1b). Accordingly, these soils are referred to as “phytohormone-conditioned” or “herbivore-conditioned,” depending on the induction method used per experiment.

In each experiment, conditioned soil from a specific treatment was thoroughly mixed in a 1:1 ratio (w/w) with γ-irradiated MiCRop soil, following guidelines for PSF experiments (Kaplan et al. 2018). For clarity, we will collectively refer to this 1:1 mixture as “soil treatment.” One-liter pots were filled with their allocated soil treatment, and sowing, germination, and growth of *B. oleracea* plants followed the same methods as in the conditioning phase. To investigate the effect of defense-conditioned soil, we challenged plants with herbivores from different feeding guilds and evaluated their performance as a proxy for plant resistance. For both the phytohormone-conditioned and herbivore-conditioned experiments, aphid infestation started 23 DAS, and caterpillar infestation 1 week later, at 30 DAS. For aphid infestation, ten 8-day-old wingless adults were transferred to the first fully developed leaf surrounded by a cotton ring to avoid movement, and left to produce nymphs. After 24 h, adult aphids were removed along with any extra nymphs until 10 neonate nymphs were kept per plant for 14 days until harvest. For caterpillar infestation, five L_1_  *M. brassicae* caterpillars were placed on the first fully developed leaf and left to feed on the plant for 18 days until harvest. The timing for herbivore infestation differed between caterpillars and aphids to reduce the infestation time of caterpillars from 14 to 8 days and avoid overfeeding of the plant. After infestation, a mesh bag was placed around each plant to contain insects until harvest, including uninfested controls. We used a completely randomized design to assign soil and herbivory treatments to plants in both the experiments. In total there were nine unique combinations of soil treatments and herbivory infestation in each experiment. Each unique combination had 10 biological replicates, with a total of 90 plants for each feedback phase (Fig. 1; Supplementary Fig. S1b).

All the plants were harvested at 37 DAS. At harvest, both herbivores were removed from plants and kept separately to measure insect performance. To measure caterpillar performance, we recovered all the caterpillars found on one plant, placed them in a single Petri dish, and weighed each caterpillar individually on a CP2P-F microbalance (Sartorius, Göttingen, Germany). Analysis was done with the average caterpillar weight per plant. Aphid performance was measured at two time points: first at 8 dpi the number of nymphs was counted on the plant and at 14 dpi we gently brushed off the aphids from each leaf to recover the total number of aphids per plant on a sticky trap to be counted later. Directly after herbivore removal, leaf discs were collected, flash-frozen, and kept at −80°C until processing to measure endogenous phytohormone and GLS levels as described in the conditioning phase. Shoots were cut at soil level and oven-dried for 3 days at 70°C to measure the dry biomass. The feedback phase from the phytohormone-treated experiment started on 27 July 2021, and the one from the herbivory-infested experiment 5 weeks later on 31 August 2021.

### Phytohormone and GLS analyses

Frozen leaf-disc samples collected at harvest from both conditioning and feedback phases were ground into a fine powder and freeze-dried using a Snijders type 2040 lyophylizer (Tilburg, the Netherlands), yielding 10–20 mg of dry leaf material. Phytohormone levels were quantified by LC–MS/MS as described in earlier studies (Karssemeijer et al. 2020, Vadassery et al. 2012), using an Agilent 1200 high-performance liquid chromatography (HPLC) system (Agilent Technologies) coupled to a QTRAP 6500 tandem mass spectrometer (SCIEX, Darmstadt, Germany). GLS were quantified by HPLC-UV according to the procedure outlined in Karssemeijer et al. (2022) and established protocols (Brown et al. 2003, Burow et al. 2006). The aliphatic GLS detected included 3-methylsulfinylpropyl glucosinolate (3MSOP), 4-methylsulfinylbutyl glucosinolate (4MSOB), and 2-propenyl-glucosinolate (Sinigrin). The indole GLS detected comprised indol-3-ylmethyl glucosinolate (I3M), 1-methoxy-indol-3-ylmethyl glucosinolate (1MOI3M), and 4-methoxy-indol-3-ylmethyl glucosinolate (4MOI3M). Total GLS concentrations for both aliphatic and indole types were calculated by summing the concentrations of the individual compounds within each group. For detailed information on phytohormone and glucosinolate methods, see Supplementary Methods S1 and S2.

### Effects of SA-conditioned soil treatment at low aphid density

To evaluate plant resistance mediated by defense-conditioned soil under low aphid infestation pressure, we grew a new set of *B. oleracea* plants in soil conditioned by SA-treated plants (SA-conditioned soil) or mock-treated plants (control-conditioned soil). These soil treatments were obtained as previously described for the phytohormone-treated experiment. Sowing, germination, and plant growth followed the same protocols detailed earlier for the feedback phase (Supplementary Fig. S6a). Each plant was infested with a single *M. persicae* aphid, and we assessed aphid feeding behavior using the EPG technique and aphid population development as proxies for plant resistance.

Aphid probing behavior was measured with EPG recording according to the adapted methodology of Kloth et al. (2021). In brief, one day before recording, 8-day-old apterous adult aphids were transferred to a *B. oleracea* leaf to acclimatize to the host plant species. On the recording day (18 DAS), an 18-*μ*m-thin gold wire of length 1.5–2 cm was gently attached to the dorsum of each aphid with water-based silver glue. Each aphid was subsequently transferred to a unique plant grown in either SA or control soil. A grounded electrode was inserted in each pot and during the course of 8 h, and electrical signals associated with stylet activities were recorded with a direct current Giga-8 system (http://epgsystems.eu; Tjallingii 1988). Both soil treatments were represented in each recording. Recordings with missing activities for more than 1 h were removed from the analysis to avoid any experimental artifacts, such as aphid escapes or wiring issues. Waveforms were annotated with the EPG Stylet software (http://epgsystems.eu).

To assess aphid population development under low-density conditions, one day after EPG recording (19 DAS), the wired adult aphid was removed, and a single newly born nymph was left on each plant to develop for 14 days in a greenhouse under the same conditions as the previous PSF experiments (Supplementary Fig. S6b). All the plants were harvested at 32 DAS, and the total number of aphids per plant was counted by brushing all of them off the leaves onto a grid paper for easier enumeration. The shoot biomass was measured as previously described for the PSF experiments.

### Root microbiome DNA extraction and amplicon sequencing

Rhizosphere DNA was extracted using the DNeasy PowerSoil Pro Kit (Qiagen) following the manufacturer’s instructions with minor modifications. Briefly, roots were weighed and subjected to chemical lysis with an in-house lysis buffer and C1 solutions (Supplementary Table S7) at 3 and 0.24 mL per gram of tissue, respectively. To facilitate the removal of soil attached to the roots, sterile 3-mm glass beads were added, followed by a 10-min incubation at 70°C and shaking in a paint shaker (SK550 1.1, Fast & Fluid, Sassenheim, the Netherlands). The extraction was then continued according to the standard protocol until the final binding and washing steps, which were automated using a KingFisher Flex instrument (Thermo Fisher) with a customized ClearMag Binding Solution/ClearMag Beads mixture (Qiagen, 22.5:1 ratio), and our in-house C5 solution (Supplementary Table S7). DNA quality and concentration were measured using a Nanodrop 2000 (ThermoFisher).

For bacteria, the V3–V4 region of the 16S rRNA gene was amplified using the primers 341f and 805r (341f: 5′- CCTACGGGNGGCWGCAG, 805r: 5′- GACTACHVGGGTATCTAATCC). For fungi, the ITS2 region was amplified using the primers ITS1f and ITS2 (ITS1F: 5′CTTGGTCATTTAGAGGAAGTAA; ITS2: 5′GCTGCGTTCTTCATCGATGC). Amplicon libraries were prepared and sequenced on a NovaSeq platform (Illumina) at Genome Quebec (Canada).

### Root microbiome community profiling

Raw FASTQ sequences were processed using the DADA2 pipeline (Callahan et al. 2016) with modifications to adjust for NovaSeq data as described in Berdaguer et al. (2024). Primer sequences were trimmed using Cutadapt (Martin 2011). After quality filtering, error correction was tailored to NovaSeq sequencing using error model number 4, following the recommendations from the Ernakovich Lab pipeline (https://github.com/ErnakovichLab/dada2_ernakovichlab). Chimeric sequences were removed, and dereplication was performed to generate ASVs following the standard DADA2 protocol.

Taxonomy was assigned using the classify-sklearn function from Qiime2 (v2022.2; Caporaso et al. 2010) with pretrained Naïve Bayes classifiers, employing the SILVA database (v138.1; Quast et al. 2012) for bacteria and the UNITE database (v8.3; Abarenkov et al. 2023) for fungi. Sequences not assigned at the phylum level were manually blasted against the NCBI database (Altschul et al. 1990), and phylogenetic trees were constructed using the align-to-tree-mafft-fasttree function in Qiime2.

For bacterial communities, ASVs identified as contaminants using the function isContaminant from the decontam package (Davis et al. 2018), as well as those assigned to mitochondria, chloroplasts, or >380 bp, were removed. For ITS data, ASVs not classified within the kingdom Fungi or those >100 bp were discarded. Samples with <10 000 reads were excluded for both bacterial and fungal datasets. This filtering resulted in an average of 89 335 reads and 10 023 ASVs for bacteria, and 111 914 reads and 466 ASVs for fungi, across both the phytohormone-treated and herbivore-infested experiments (see Supplementary Table S8 for details).

### Statistical analysis

All the statistical analyses were performed in R (v4.4.0, R Core Team, 2024) using RStudio (v4.1.1748). Microbiome and plant-trait analyses were performed separately per induction method unless noted. Alpha diversity metrics (species richness, Shannon index, and Faith’s phylogenetic diversity) were calculated with the estimate_richness function (phyloseq package; McMurdie and Holmes 2013) on median-transformed normalized counts to account for differences in sequencing depth (Supplementary Table S3). Each metric was analyzed with a GLM using stress treatment as the main factor. For beta diversity analyses, ASVs with a relative abundance below 0.0001% or detected in fewer than 10% of bacterial samples, or fewer than 5% of fungal samples, were excluded. Counts were normalized using cumulative-sum scaling (CSS) transformation (metagenomeSeq package; Paulson et al. 2013), and UniFrac distances were calculated using phyloseq. Differences in community composition between induction treatments were assessed using PERMANOVA with the adonis2 function (vegan package; Oksanen et al. 2022) for both combined and separate experiments. Results were visualized with Unifold Manifold Approximation and Projection (UMAP) plots (Konopka 2023).

Defense-responsive ASVs were identified by differential abundance analysis between JA and SA induction treatments and controls using the ancombc function (ANCOM-BC package; Lin and Peddada 2020) with structural zeroes and the Benjamini–Hochberg false discovery rate (FDR) correction with a significance threshold of *P*_PDR_ < 0.05. ASVs present in <20% of bacterial samples or <10% of fungal samples were filtered out before analysis and log-fold changes (LFCs) between induction treatments and controls were calculated. To generate a better-resolved phylogeny of the defense-responsive ASVs, a multiple sequence alignment was created for bacterial sequences and for fungal sequences using MAFFT v7 using the iterative refinement method FFT-NS-i (Katoh et al. 2019). The best-fitting substitution model was determined using IQ-TREE (Nguyen et al. 2015), and maximum likelihood phylogenetic inference was performed with 10 000 bootstrap replicates. Final microbial phylogenetic trees were annotated in iTOL (Letunic and Bork 2021).

GLMs (with Gaussian, gamma, or Poisson families as appropriate) were used to assess the effects of induction treatments on plant traits, herbivore performance, and other variables. Model assumptions were validated using the simulateResiduals function (DHARMa package; Hartig 2024), and *post hoc* comparisons were conducted using a Tukey least squares discriminant (LSD) test with the emmeans function (*emmeans* package; Lenth 2024). For the conditioning phase, plant traits (biomass, aliphatic GLS, and indole GLS) were analyzed with induction treatment as the main factor. In the feedback phase, soil treatment, herbivory type, and their interaction were included. Endogenous phythormone levels were assessed by a PLS-DA, retaining only phytohormones with a VIP score > 0.75 for the final models. A PERMANOVA with Euclidean distances was then used to test the effects of soil treatment, herbivory type, and induction method on phytohormonal profiles. Caterpillar weight at 8 dpi was analyzed with soil treatment as the main factor, while aphid counts were analyzed with soil treatment, time point, and their interaction (high-density experiment) or soil treatment alone (low-density experiment).

EPG waveforms were processed following Kloth et al. (2021). Stylet activities that were interrupted by the end of the recording were included in the analyses. Waveforms not performed by aphids were treated as missing values for mean and maximum duration and as the end time of the recording for latency analyses. EPG summary variables were analyzed with Mann–Whitney *U* pairwise comparisons, except for proportional variables, such as percentages, which were analyzed with an *X*^2^ test. Time-trend analyses of the percentage of time spent in the pathway phase and phloem feeding were performed using a GLMM with soil treatment, recording hour, and their interaction as main factors, and aphid/plant individual as random factor to correct for any bias between individual aphids. The code used for all the analyses is available on GitHub at https://github.com/Marcelara/PSF_JAandSA.

## Supplementary Material

Supplementary_materials_pcag048

## Data Availability

Raw sequencing data are available at NCBI BioProject accession numbers PRJNA1217616 (16S) and PRJNA1217804 (ITS). The data underlying this article are available on GitHub at https://github.com/Marcelara/PSF_JAandSA.
