# Supplementary material for: Jasmonic and salicylic acid pathways shape the rhizosphere microbiome, affecting aphid herbivory and soil-mediated insect–plant interactions in a density-dependent manner
Source: Plant Cell Physiol. 2026 Apr 14;67(9):1473–88. doi: 10.1093/pcp/pcag048 (PMC13621220; doi:10.1093/pcp/pcag048)
Supplement: Supplementary file 1 [file supplementary_materials_pcag048.pdf]

Supplementary Information to manuscript:

"Jasmonic and Salicylic Acid Pathways Shape the Rhizosphere Microbiome, affecting Aphid Herbivory and Soil-Mediated Insect-Plant Interactions in a density-dependent manner"

Marcela Aragón[1], Haris Spyridis[1], Pieter Mostard[1], Michael Reichelt[2], Jonathan Gershenzon[2], Marcel Dicke[1] and Karen J. Kloth[1#].

[1] Laboratory of Entomology, Wageningen University & Research, Wageningen, The Netherlands.

[2] Department of Biochemistry, Max Planck Institute for Chemical Ecology, Jena, Germany.

[#]Corresponding auhor: karen.kloth@wur.nl

The Following Supplementary Information is available:

Supplementary Figures **3**

Supplementary Figure S1. Scheme of PSF experimental setup with high aphid density. 4

Supplementary Figure S2. Individual endogenous phytohormone levels in the conditioning phase. 5

Supplementary Figure S3. Alpha diversity metrics of bacterial communities across both experiments. 6

Supplementary Figure S4. Alpha diversity metrics of fungal communities across both experiments. 7

Supplementary Figure S5. Total aliphatic and indole glucosinolate (GLS) content of Brassica oleracea leaves in the feedback phase. 8

Supplementary Figure S6. Scheme of low-aphid density PSF experimental setup. 9

Supplementary Tables **10**

Supplementary Table S1. Results of Generalized Linear Models (GLMs) testing the effects of treatment on plant variables in the conditioning phase. 10

Supplementary Table S2. PERMANOVA results on endogenous phytohormone profiles during conditioning and feedback phases. 11

Supplementary Table S3. Results of Generalized Linear Models (GLMs) testing the effects of soil treatment, herbivory and their interactions on plant and insect variables in the feedback phase. ... 12

Supplementary Table S4. Parameters of *Myzus persicae* feeding behavior (mean ± SD) measured during 8-hr electrical penetration graph recordings on *Brassica oleracea* plants grown in control- or SA-conditioned soil. 13

Supplementary Table S5. Results of Generalized Linear Models (GLMs) and Generalized Linear Mixed Models (GLMMs) testing the effects of soil inocula on plant biomass, aphid feeding behavior and reproduction feedback phase of the low-density experiment. 14

Supplementary Table S6. Measured values of essential macro- and micro-nutrients, and chemical, physical and biological properties of MiCRop soil. 15

Supplementary Table S7. Recipes for in-house DNA extraction buffers used in this study. 16

Supplementary Table S8. Number of reads and ASVs retained for 16S and ITS communities after filtering across experiments and treatments. 17

Supplementary Methods **18**

Supplementary Methods S1. Quantification of Phytohormones by LC-MS/MS 18

Supplementary Methods S2. Glucosinolate analysis by HPLC-UV 19

Supplementary Figures

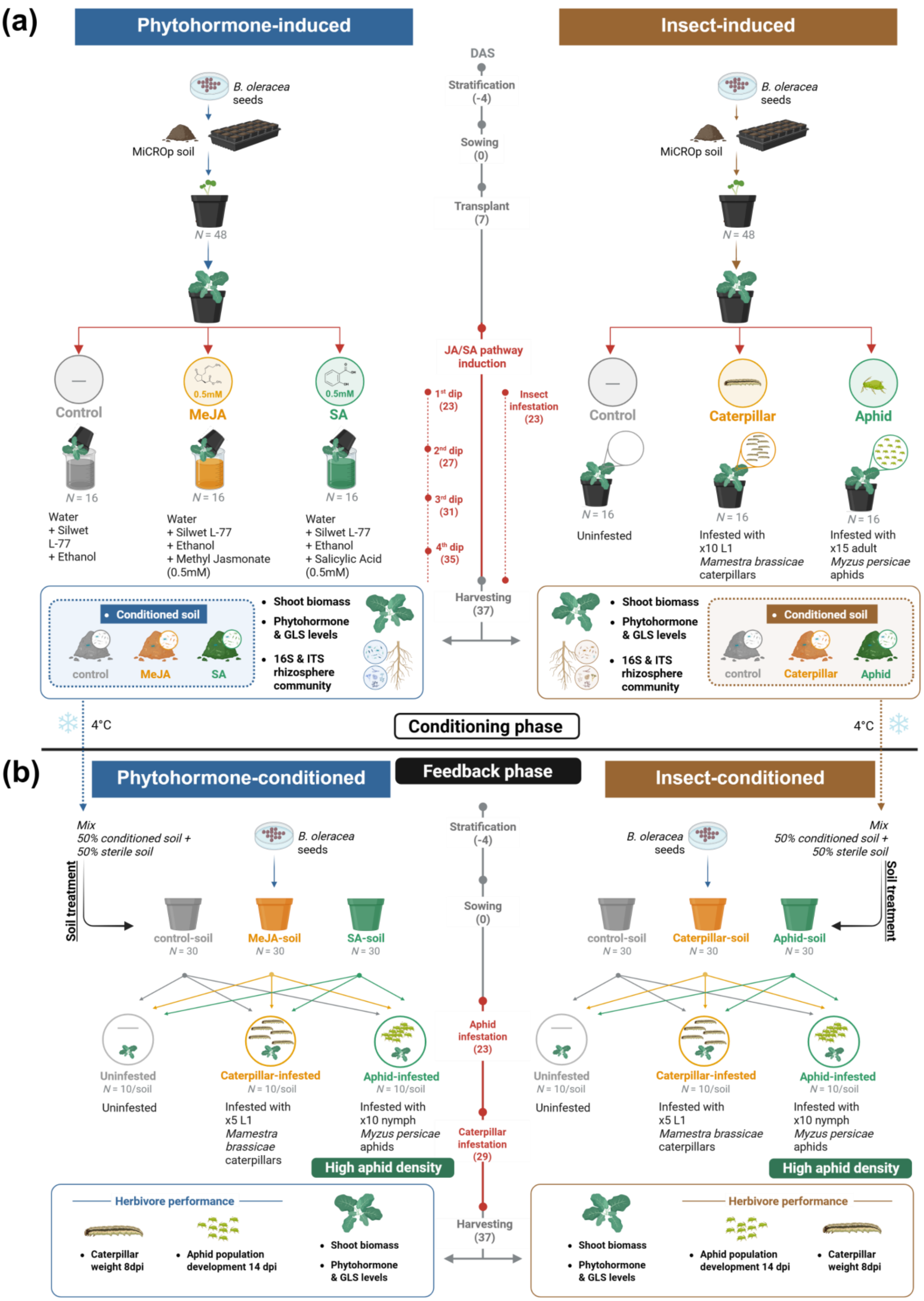

(Figure legend on next page)

Supplementary Figure S1. Scheme of PSF experimental setup with high aphid density.

(a) In the conditioning phase, seeds of *Brassica oleracea* were stratified, germinated and transplanted as individual plants into pots containing the same MiCROp soil background. This phase was designed to induce defense pathway-specific changes in the rhizosphere microbiome. Plants were exposed to one of three induction treatments 23 days after sowing (DAS), depending on the type of experiment (Phythormone-induced or Herbivore-induced). At harvest (37 DAS), leaf discs were taken from the shoots to measure phytohormone and glucosinolate levels, and the rest of the shoots were clipped to measure plant biomass. Additionally, roots were processed for 16S and ITS rhizosphere amplicon sequencing. All soil from pots within each stress treatment was pooled, thoroughly mixed, and stored at 4◦C as conditioned soil to be used in the feedback phase. **(b)** The feedback phase was designed to test the effect of defense-conditioned soils in plant growth and resistance to insect herbivory. For this, conditioned soils were mixed in a 1:1 ratio with sterilized soil to create soil treatments. New seeds of *B. oleracea* were stratified and sown into individual pots containing the allocated soil treatment. For each soil treatment plants were assigned to one of three herbivory conditions: aphid infestation, caterpillar infestation or uninfested controls. Aphid infestation started at 23 DAS with a high initial density of 10 nymphs per plant, while caterpillar infestation began at 29 DAS. Aphid performance was evaluated by counting the total number of nymphs at 8 and 14 days post-infestation (dpi). Caterpillar performance was assessed by weighing caterpillars at 8 dpi. Plants were harvested at 37 DAS, and shoots were processed as described for the conditioning phase. Numbers in between brackets indicate the number of DAS. Figure created in https://BioRender.com

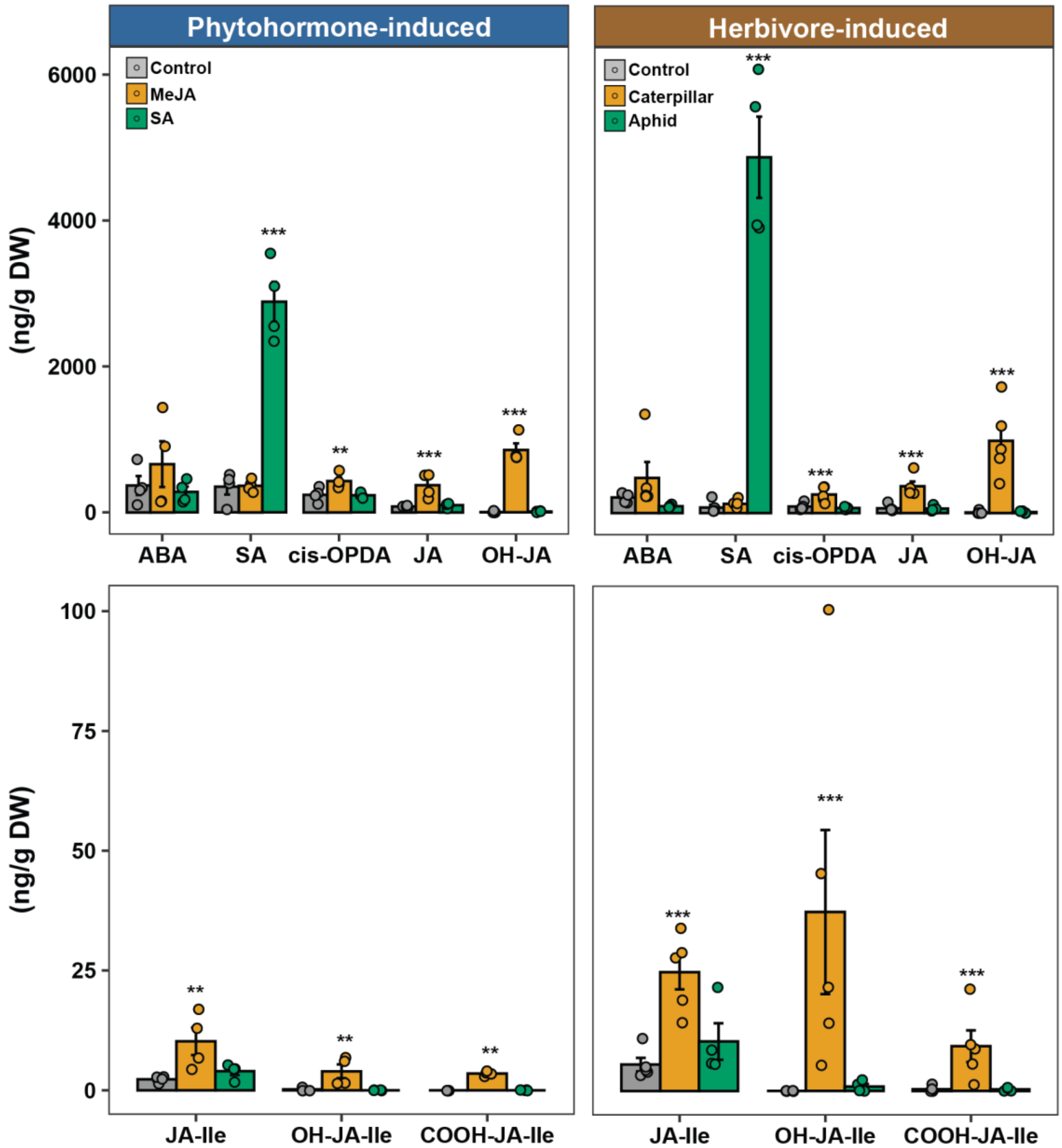

Supplementary Figure S2. Individual endogenous phytohormone levels in the conditioning phase.

The phytohormones analyzed included: Abscisic acid (ABA), Salicylic Acid (SA), the jasmonate precursor 12-Oxo-Phytodienoic Acid (cis-OPDA), Jasmonic Acid (JA), JA catabolite Hydroxyjasmonic Acid (OH-JA), bioactive Jasmonoyl isoleucine (JA-Ile), and its catabolites Hydroxyjasmonoyl isoleucine (OH-JA-Ile) and Carboxyjasmonoyl isoleucine (COOH-JA-Ile). Bars show mean +/- standard error (SE). Circles represent raw data of individual plants. Colors denote induction treatments, and experiments are displayed side by side for comparison. Data was analyzed individually per phytohormone with a Generalized Linear Model using treatment as a fixed factor with either Gamma or Negative binomial distribution as it was fit. Asterisks show the level of statistical significance differences at α=0.05 according to Tukey's LSD between each of the stress-induced treatments and controls per phytohormone (***P* < 0.01,*** *P* < 0.001). Each plant represented one biological replicate (*N* = 4-5).

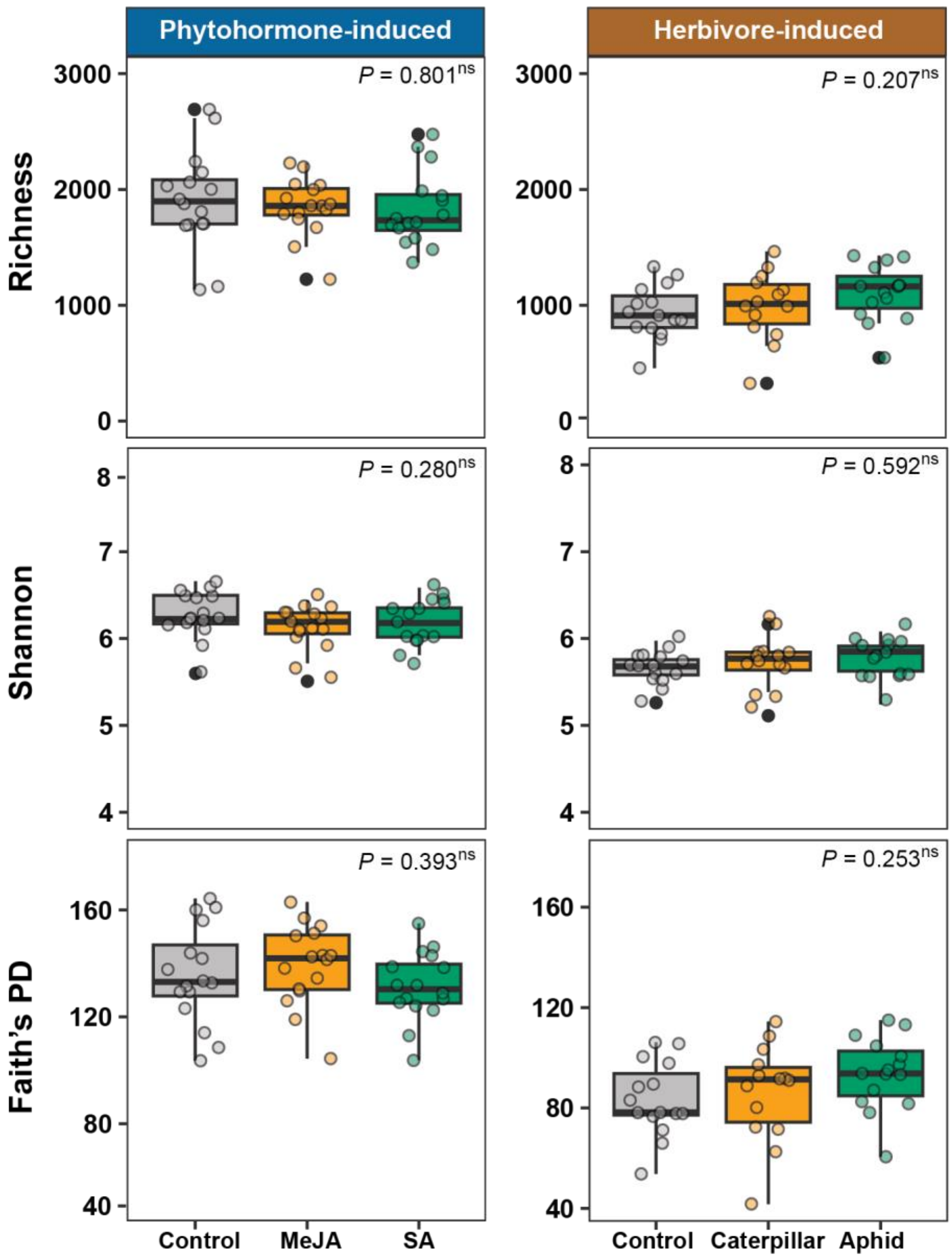

Supplementary Figure S3. Alpha diversity metrics of bacterial communities across both experiments.

Boxplots display observed species richness, Shannon diversity index, and Faith's phylogenetic diversity (PD) for rhizosphere bacterial communities in plants treated with phytohormones or infested with herbivores, across both experiments. Boxes represent the interquartile range (IQR), center lines depict the median values, and whiskers represent data within 1.5x IQR. Circles represent raw data of individual plants. Colors denote stress treatments, and experiments are displayed side by side for comparison. Data were analyzed with a Linear Model (LM) or with a Generalized Linear Model (GLM) depending on data distribution. P value and statistical significance for induction treatment is indicated as follows: ns ($P > 0.05$).

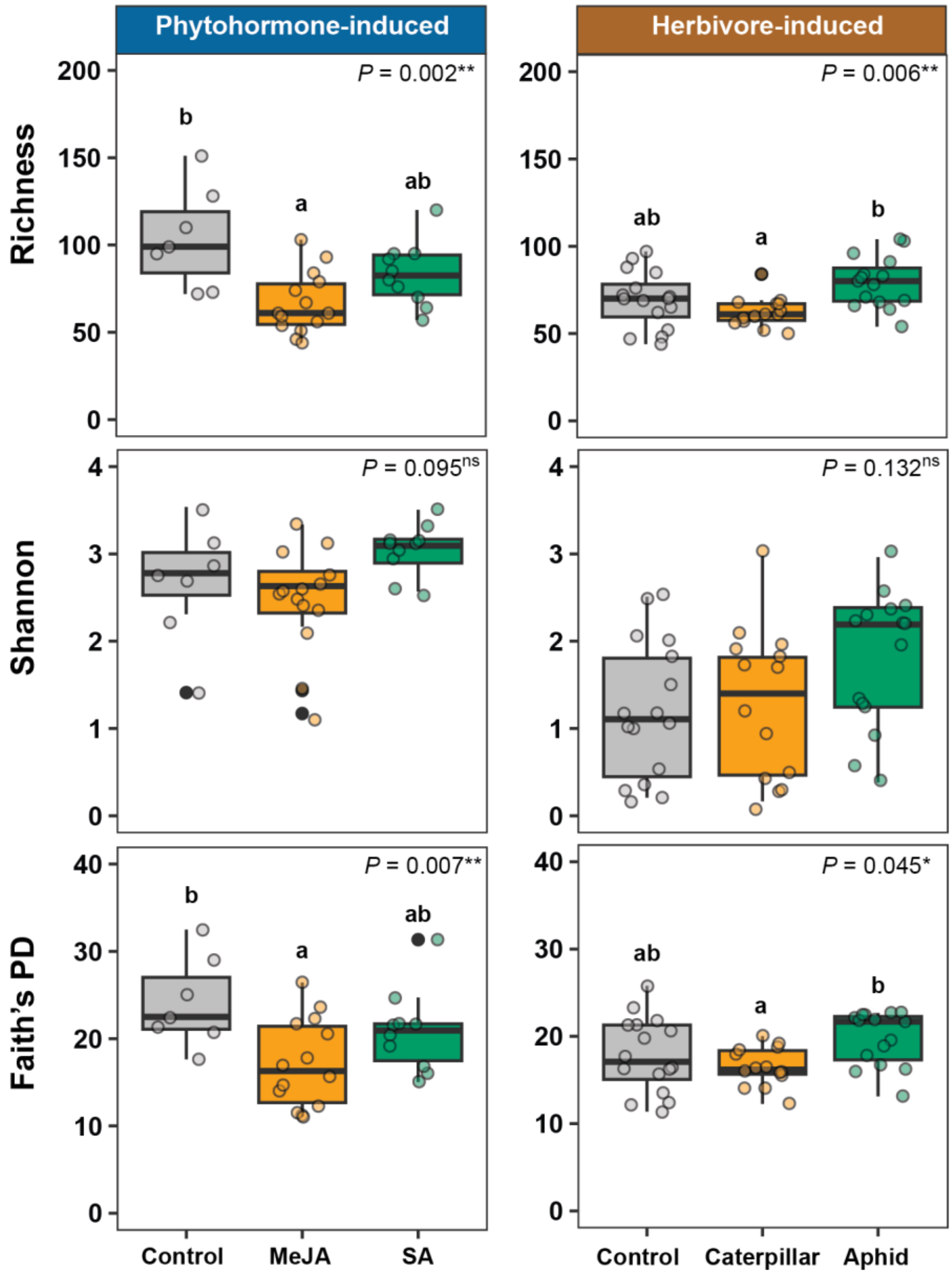

Supplementary Figure S4. Alpha diversity metrics of fungal communities across both experiments.

Boxplots display observed species richness, Shannon diversity index, and Faith's phylogenetic diversity (PD) for rhizosphere fungal communities in plants treated with phytohormones or infested with herbivores, across both experiments. Boxes represent the interquartile range (IQR), center lines depict the median values, and whiskers represent data within 1.5x IQR. Circles represent raw data of individual plants. Colors denote stress treatments, and experiments are displayed side by side for comparison. Different letters above the boxes indicate statistically significant differences between treatments ($P < 0.05$). Data were analyzed with a Linear Model (LM) or with a Generalized Linear Model (GLM) depending on data distribution. P value and statistical significance for induction treatment is indicated as follows: $^{ns}P > 0.10$, $^{\dagger}P \leq 0.10$, $^{*}P \leq 0.05$, $^{**}P \leq 0.01$.

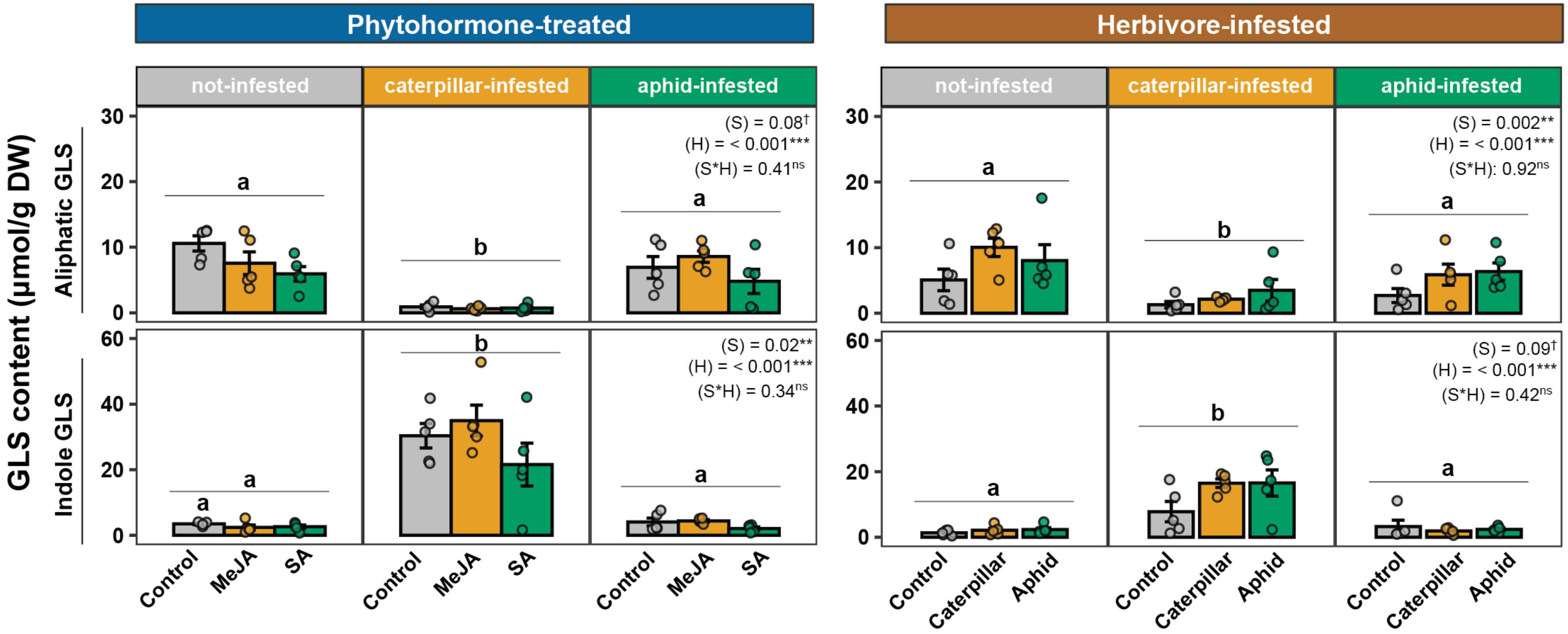

Supplementary Figure S5. Total aliphatic and indole glucosinolate (GLS) content of Brassica oleracea leaves in the feedback phase.

Barplots show the mean ± standard error (SE), circles represent raw data of individual plants. Colors denote soil treatments, and experiments are displayed side by side for comparison. Different letters above indicate statistically significant differences between herbivory as main factor ($P < 0.05$). Data were log-transformed to meet model assumptions and analyzed with a Generalized Linear Model with Gaussian family. P value and statistical significance for soil treatment (S), herbivory (H) and its interaction (S*H) are indicated as follows: $^{ns}P > 0.10$, $^{\dagger}P \leq 0.10$, $^{**}P \leq 0.01$, $^{***}P \leq 0.001$.

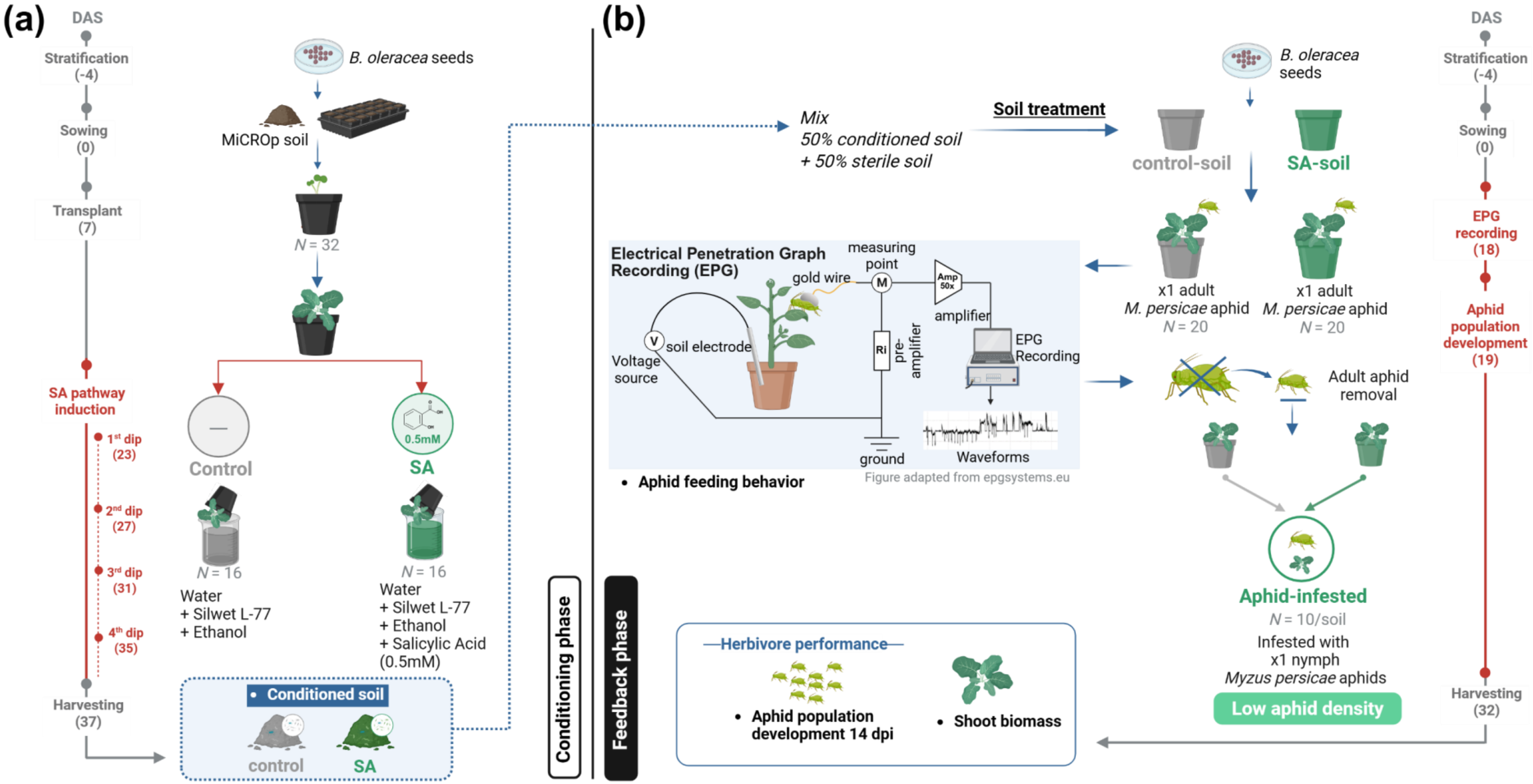

Supplementary Figure S6. Scheme of low-aphid density PSF experimental setup.

(**a**) In the conditioning phase, seeds of *Brassica oleracea* were stratified, germinated, and sown into pots containing MiCROp soil. Plants were exposed to one of two induction treatments at 23 days after sowing (DAS): Salicylic Acid (SA) or Mock-treated control. Treatments were applied to the aboveground lead area by dipping plants in solution containing 0.5mM SA, Silwet and ethanol or in a mock solution containing only Silwet and ethanol. Defense induction lasted for 14 days, with treatments applied every 3 days (4 applications in total). At harvest (37 DAS), soil from all pots in each treatment was pooled, homogenized, and stored at 4°C as conditioned soil for the feedback phase. **(b)** In the feedback phase, soil treatments were prepared by mixing SA-conditioned soil with sterilized

MiCROp soil in a 1:1 ratio. New seeds of *B. oleracea* seeds were stratified, germinated and grown in individual pots containing the allocated soil treatment. At 18 DAS, *Myzus persicae* aphids were wired with a fine gold wire to measure aphid feeding behavior using the Electrical Penetration Graph (EPG) technique. After wiring, one adult aphid was placed on each plant to record stylet activities for 8 hours. Following the EPG recording, the adult aphid was removed, and a single newly born nymph was left on each plant to develop for 14 days. At harvest (32 DAS), the number of aphids per plant was recorded, and plant biomass was measured. Numbers in between brackets indicate the number of DAS. Figure created in https://BioRender.com

Supplementary Tables

Supplementary Table S1. Results of Generalized Linear Models (GLMs) testing the effects of treatment on plant variables in the conditioning phase.

				Phytohormone-induced			Herbivore-induced		
**Variable**	**Model type**	**Model**	**Factor(s)**	**X2**	**df**	**P-value**	**X2**	**df**	**P-value**
**Plant biomass**	GLM (Gaussian)	~Induction treatment	Treatment (T)	31.400	2	<0.001***	30.563	2	<0.001***
**Aliphatic GLS**	GLM (Gaussian-Log)	~Induction treatment	Treatment (T)	0.097	2	0.9523^{ns}	10.924	2	0.004**
**Indole GLS**	GLM (Gaussian-Log)	~Induction treatment	Treatment (T)	24.782	2	<0.001***	37.537	2	<0.001***

Note: GLMs were fitted with the specified distribution families and link functions in between brackets. P value and statistical significance are indicated as follows: ^{ns}(P > 0.05), **(P ≤ 0.01), ***(P ≤ 0.001). GLS stands for Glucosinolate.

Supplementary Table S2. PERMANOVA results on endogenous phytohormone profiles during conditioning and feedback phases.

Phase	Phytohormones with VIP > 0.75	model	Factor	df	SumOfSqs	R^2	F	P-value
Conditioning	SA, JA, ABA, cis-OPDA, OH-JA	~Experiment*Induction treatment	Experiment (E)	1	10.21	0.081	5.51	0.003**
			Treatment (T)	2	75.59	0.605	20.42	0.001**
			E*T	2	2.18	0.017	0.59	0.762^{ns}
			Residual	20	37.02	0.296		
			Total	25	125.00	1.000		
Feedback	SA, JA, cis-OPDA, OH-JA	~Experiment+Soil treatment+Herbivory	Experiment (E)	1	32.72	0.048	13.59	0.001**
			Soil treatment (S)	2	14.12	0.021	2.93	0.006**
			Herbivory (H)	2	235.82	0.347	48.96	0.001**
			Residual	165	397.34	0.584		
			Total	170	680.00	1.000		

Note: Phytohormones with VIP > 0.75 = only pytohormones included in the final model, df = degrees of freedom, SumOfSq = Sum of Squares, R^2= explained variance, F = F-statistics. *P* value and statistical significance are indicated as follows: ^{ns}($P > 0.05$), **($P \leq 0.01$), ***($P \leq 0.001$).

Supplementary Table S3. Results of Generalized Linear Models (GLMs) testing the effects of soil treatment, herbivory and their interactions on plant and insect variables in the feedback phase.

Variable	Model type	Model	Factor(s)	Phytohormone-conditioned			Herbivore-conditioned		
				X^2	df	P-value	X^2	df	P-value
**Plant biomass**	GLM (Gaussian-Log)	~ Soil treatment*Herbivory	Soil treatment (S)	3.477625	2	0.176^{ns}	2.336249	2	0.311^{ns}
			Herbivory (H)	1.595769	2	0.450^{ns}	5.68977	2	0.058†
			S*H	6.794492	4	0.147^{ns}	2.982226	4	0.561^{ns}
**Aliphatic GLS**	GLM (Gaussian-Log)	~ Soil treatment *Herbivory	Soil treatment (S)	4.967487	2	0.083†	12.71729	2	0.002**
			Herbivory (H)	110.2586	2	<0.001***	26.29864	2	<0.001***
			S*H	4.000585	4	0.406^{ns}	0.944024	4	0.918^{ns}
**Indole GLS**	GLM (Gaussian-Log)	~ Soil treatment *Herbivory	Soil treatment (S)	8.158443	2	0.017**	4.891475	2	0.087†
			Herbivory (H)	127.5123	2	<0.001***	56.56236	2	<0.001***
			S*H	4.528852	4	0.339^{ns}	3.903132	4	0.419^{ns}
**Caterpillar weight 8dpi**	GLM (Gaussian)	~ Soil treatment	Soil treatment (S)	1.369621	2	0.504^{ns}	0.731616	2	0.694^{ns}
**Aphid number**	GLM (Gamma-Log)	~ Soil treatment *Time point	Soil treatment (S)	5.679	2	0.058†	2.761	2	0.251^{ns}
			Time point(T)	141.359	1	<0.001***	96.415	1	<0.001***
			S*T	0.019	2	0.990^{ns}	0.043	2	0.979^{ns}

Note: GLMs were fitted with the specified distribution families and link functions in between brackets. *P* value and statistical significance are indicated as follows: ^{ns}($P > 0.10$), †($P \le 0.10$), **($P \le 0.01$), ***($P \le 0.001$).

Supplementary Table S4. Parameters of *Myzus persicae* feeding behavior (mean ± SD) measured during 8-hr electrical penetration graph recordings on *Brassica oleracea* plants grown in control- or SA-conditioned soil.

Behaviour		Control	SA	sign
**Pathway**	NP	50.6 ± 10.9	48.9 ± 7.1	ns
**(C)**	#	32.7 ± 5.6	37.1 ± 6.3	ns
	# short	17.7 ± 3.6	17 ± 4.5	ns
	Total	206.4 ± 22.5	286.2 ± 21.1	*P* = 0.018 *
**Salivation in phloem**	Lat	125.9 ± 22.7	103.2 ± 21.2	ns
**(E1)**	#	10.7 ± 1.5	13.5 ± 1.6	ns
	Total	25.3 ± 7	17.8 ± 2.5	ns
	Prop (%)	14.6 ± 3.5	14.3 ± 3.1	ns
	Aphids	100%	100%	ns
	sing E1	72%	93%	ns
	# sing E1	2.7 ± 0.8	3.5 ± 0.7	ns
**Phloem ingestion**	Aphids	100%	93%	ns
**(E2)**	#	6.1 ± 1	6.7 ± 1	ns
	Total	145.7 ± 30.7	88 ± 22.7	ns
	Mean	36.5 ± 10.3	12.5 ± 3.4	ns
	Max	85.6 ± 18.2	50.1 ± 13.1	ns
**Sustained phloem ingestion**	Aphids	89%	67%	ns
**(E2s)**	#	2 ± 0.4	1.5 ± 0.4	ns
	Total	134.1 ± 31.1	74.2 ± 22.8	ns
**Penetration difficulties**	Aphids	44%	27%	ns
**(F)**	Total	30.8 ± 11.5	13.4 ± 7.7	ns
**Xylem ingestion**	Aphids	44%	53%	ns
**(G)**	Total	20.8 ± 8.4	25.8 ± 9.5	ns

Note: NP = total duration non probing, # = number of times probe was detected during recording, # short = pathway probes < 3 minutes, Total = total duration pathway (min), Lat = latency from the first probe of the recording to the first occurrence (min), Prop (%) = proportion of time spent on salivation relative to the total phloem phase, Aphids = percentage of aphids performing the behavior, sing E1 = number of single salivations that are not directly preceded or followed by phloem ingestion, Mean = mean duration (min), Max = maximum duration (min). Data show mean ± SD. *N* = 15 for Control, and *N* = 18 for SA. sign = significance, ns = not-significant. Proportional variables (%) were tested with a X^2 test and other variables were tested with Mann-Whitney U pairwise comparison tests.

Supplementary Table S5. Results of Generalized Linear Models (GLMs) and Generalized Linear Mixed Models (GLMMs) testing the effects of soil inocula on plant biomass, aphid feeding behavior and reproduction feedback phase of the low-density experiment.

Variable	Model type	Model	Factor(s)	LR Chisq	df	P-value
**Aphid number 14dpi**	GLM (Gamma-Log)	~ Soil treatment	Soil treatment (S)	8.80	1	0.003**
**Biomass**	GLM (Gaussian)	~ Soil treatment	Soil treatment (S)	1.7639	1	0.1841ns
**Phloem feeding**	GLMM (Poisson)	~ Soil treatment *Time + (1 | Plant ID)	Soil treatment (S)	0.414	1	0.233ns
			Time (T)	812.38	1	<0.001***
			S*T	135.54	7	<0.001***

Note: GLMs were fitted with the specified distribution families and link functions in between brackets.

P value and statistical significance are indicated as follows: ns(P > 0.10), †(P ≤ 0.10), *(P ≤ 0.05), **(P ≤ 0.01), ***(P ≤ 0.001).

Supplementary Table S6. Measured values of essential macro- and micro-nutrients, and chemical, physical and biological properties of MiCRop soil.

Category	Nutrient	Value	Unit
**Macronutrients**	Nitrogen (N)	940	mg N/kg
	Sulfur (S)	4	mg S/kg
	Total Sulfur (S)	220	mg S/kg
	Phosphorus (P)	0.5	mg P/kg
	Total Phosphorus (P_2O_5)	42	mg P2O5/100g
	Potassium (K)	37	mg +/kg
	Calcium (Ca)	0.5	mmol +/l
	Total Calcium (Ca)	33	mmol Ca/kg
**Micronutrients**	Magnesium (Mg)	61	mg +/kg
	Total Magnesium (Mg)	4.3	mmol Mg/kg
	Sodium (Na)	<6	mg +/kg
	Silicon (Si)	6270	µg Si/kg
	Iron (Fe)	<2010	µg Fe/kg
	Zinc (Zn)	500	µg Zn/kg
	Manganese (Mn)	2620	µg Mn/kg
	Copper (Cu)	31	µg Cu/kg
	Cobalt (Co)	6	µg Co/kg
	Boron (B)	92	µg B/kg
	Molybdenum (Mo)	<4	µg Mo/kg
	Selenium (Se)	<2.1	µg Se/kg
**Chemical properties**	pH	5.4	-
	Organic Carbon (C)	1.8	%
	Organic Matter	3.2	%
	Inorganic Carbon (C)	0.06	%
	Calcium Carbonate (CO_3)	<0.2	%
**Physical properties**	Clay (<2 µm)	2	%
	Silt (2-50 µm)	11	%
	Sand (>50 µm)	84	%
**Cation Exchange Capacity**	CEC (Cation Exchange Capacity) - clay-humus	33	mmol +/kg
**Biological Properties**	Microbial Biomass	23	mg C/kg
	Microbial Activity	7	mg N/kg
	Fungi:Bacteria ratio	0.4	-

Supplementary Table S7. Recipes for in-house DNA extraction buffers used in this study.

Solution	Recipe
**Lysis buffer**	Mix filter-sterilized (0.2 μm filter) 605 mM Guanidine thiocyanate with sterile 362 nM sodium dihydrogen phosphate in 1:1 ratio. Adjust pH to 8-8.5, dilute to 1L with Mili-Q® purified water
**C1**	Mix 50 mL of filter-sterilized 8% of Sodium dodecyl sulfate (SDS), 10 mL of 1.5 M sodium chloride and 33.33 mL of Tris (both sterile and with adjusted pH to 8-8.5). Dilute to 100 mL with sterile Mili-Q® water.
**C5**	Combine 0.1 mL of Tris and 1.3 mL of 1.5 M sodium chloride (both sterile and with adjusted pH to 8-8.5), and 10.4 mL of absolute ethanol. Dilute to 20 mL with sterile Mili-Q® water.
**Elution buffer**	Sterile 10 mM Tris (pH 8-8.5) prepared in sterile Mili-Q® water.

Supplementary Table S8. Number of reads and ASVs retained for 16S and ITS communities after filtering across experiments and treatments.

Community	Experiment	Treatment	reads kept (%)	reads/sample	number of ASVs	*N*
16S	Phytohormone-induced	Control	77.8 ± 13.8	125,138 ± 64,878	14,433	16
		MeJA	81.5 ± 12.7	91,376 ± 25,641	12,857	16
		SA	79.3 ± 10.8	123,129 ± 71,242	13,484	16
	Herbivore-induced	Control	84.3 ± 12.9	59,651 ± 25,224	6,193	15
		Caterpillar	91.7 ± 2.62	65,834 ± 21,386	6,293	15
		Aphid	91.5 ± 6.83	70,883 ± 17,915	6,877	14
ITS	Phytohormone-induced	Control	98.6 ± 1.03	112,307 ± 42,948	425	7
		MeJA	96.6 ± 4.24	121,096 ± 27,327	451	14
		SA	98.7 ± 1.33	113,026 ± 17,748	446	10
	Herbivore-induced	Control	99.2 ± 1.48	97,987 ± 19,237	484	16
		Caterpillar	96.9 ± 9.65	110,827 ± 22,998	438	14
		Aphid	98.8 ± 1.25	116,240 ± 16,538	555	15

Note: Community: Microbial community analyzed (16S for bacteria and ITS for fungi), reads kept (%) = percentage from total reads retained after filtering steps (mean ± SD), reads/sample = average number of reads per sample after filtering steps (mean ± SD), number of ASVs = total number of ASVs for each treatment group, N = final number of samples retained after filtering in each treatment group.

Supplementary Methods

Supplementary Methods S1. Quantification of Phytohormones by LC-MS/MS

Lyophilized plant material (10-20 mg) were extracted and homogenized in 1 ml methanol containing 60 ng D4-SA (Santa Cruz Biotechnology, USA), 60 ng D6-JA (HPC Standards GmbH, Germany), 60 ng D6-ABA (Toronto Research Chemicals, Toronto, Canada), and 12 ng D6-JA-Ile (HPC Standards GmbH) as internal standards. Samples were agitated on a horizontal shaker at room temperature for 10 min. The homogenate was mixed for 30 min and centrifuged at 13,000 rpm for 20 min at 4 °C and the supernatant was collected. Phytohormone analysis was performed as in Vadassery et al (2012) and Karssemeijer et al. (2022) on an Agilent 1200 series HPLC system (Agilent Technologies) coupled to a tandem mass spectrometer QTRAP 6500 (SCIEX, Darmstadt, Germany). Separation of raw extracts (see above) was achieved on a Zorbax Eclipse XDB-C18 column (50 × 4.6 mm, 1.8 mm; Agilent) with a solvent system of 0.05% formic acid (A) and acetonitrile (B) at a flow rate of 1.1 ml/min. The elution profile was the following: 0 to 0.5 min, 10% B; 0.5 to 4.0 min, 10% to 90% B in A; 4.0 to 4.02 min, 90% to100% B, 4.02 to 4.5 min, 100% B and 4.51 to 7.0 min 10% B. The column temperature was maintained at 25°C. The mass spectrometer was equipped with a Turbo spray ion source operated in negative ionization mode. The ion spray voltage was maintained at -4,500 eV. The turbo gas temperature was set at 650°C. Nebulizing gas was set at 60 psi, curtain gas at 40 psi, heating gas at 60 psi, and collision gas was set to "medium". The mass spectrometer was operated in multiple reaction monitoring (MRM) mode, details of the instrument parameters and response factors for quantification can be found in Table M1. Since we observed that both, the D6-labeled JA and D6-labeled JA-Ile standards (HPC Standards GmbH, Cunnersdorf, Germany) contained 40 % of the corresponding D5-labeled compounds, the sum of the peak areas of D5- and D6-compound was used for quantification.

Table M1. Details of analysis of phytohormones and plumbagin by LC-MS/MS [HPLC 1260 (Agilent Technologies)-QTRAP6500 (SCIEX)] in negative ionisation mode. Q1 and Q3: selected m/z of the first and third quadrupole, respectively; RT: retention time; RF: Response factor; DP: declustering potential; CE: collision energy.

Q1	Q3	RT (min)	Compound	Internal standard	RF	DP	CE
136.93	93.00	3.3	SA	D4-SA	1.0	-20	-24
263.00	153.20	3.4	ABA	D6-ABA	1.0	-20	-22
209.07	59.00	3.6	JA	D6-JA+D5-JA	1.0	-20	-24
322.19	130.10	3.9	JA-Ile	D6-JA-Ile+D5-JA-Ile	1.0	-50	-30
290.90	165.10	4.6	OPDA	D6-JA+D5-JA	1.0	-20	-24
338.10	130.10	3	OH-JA-Ile	D6-JA-Ile+D5-JA-Ile	1.0	-50	-30
225.10	59.00	2.6	OH-JA	D6-JA+D5-JA	1.0	-20	-24
352.10	130.10	3	COOH-JA-Ile	D6-JA-Ile+D5-JA-Ile	1.0	-50	-30
140.93	97.00	3.3	D4-SA			-20	-24
269.00	159.20	3.4	D6-ABA			-20	-22
215.00	59.00	3.6	D6-JA			-20	-24
214.00	59.00	3.6	D5-JA			-20	-24
328.19	130.10	3.9	D6-JA-Ile			-50	-30
327.19	130.10	3.9	D5-JA-Ile			-50	-30

Supplementary Methods S2. Glucosinolate analysis by HPLC-UV

Glucosinolates were analysed by HPLC-UV as described in Burow *et al.* (2006) and Karssemeijer *et al.* (2022). 6-25 mg of lyophilized root material were extracted with 1mL methanol solution (v:V) containing 50nmol of 4-hydroxybenzyl glucosinolate. A 500uL aliquot of the raw extract was loaded onto DEAE Sephadex A 25 columns and treated with arylsulfatase for desulfation (Sigma-Aldrich) (Brown *et al.* 2003). The eluted desulfoglucosinolates were separated using high performance liquid chromatography (Agilent 1100 HPLC system, Agilent Technologies) on a reversed phase C-18 column (Nucleodur Sphinx RP, 250 x 4.6 mm, 5µm, Machrey-Nagel, Düren, Germany) with a water (A)-acetonitrile (B) gradient (0-1 min, 1.5% B; 1-6 min, 1.5-5% B; 6-8 min, 5-7% B; 8-18 min, 7-21% B; 18-23 min, 21-29% B; 23-23.1 min, 29-100% B; 23.1-24min 100% B and 24.1-28 min 1.5% B; flow 1.0 mL min-1). Detection was performed with a photodiode array detector and peaks were integrated at 229 nm. Desulfated glucosinolates were identified by comparison of retention time and UV spectra to those of purified standards previously extracted from Arabidopsis thaliana (Brown *et al*. 2003) or by analysis of the desulfoglucosinolate extracts on an LC-ESI-Ion-Trap-mass spectrometer (Esquire6000, Bruker Daltonics). We used the following molar response factors for quantification of individual Gls relative to the internal standard, 4-hydroxybenzyl glucosinolate: aliphatic Gls 2.0, indole Gls 0.5 (Burow *et al*. 2006). The following glucosinolates were detected in the samples: 3-methylsulfinylpropyl glucosinolate (3MSOP), 4-methylsulfinylbutyl glucosinolate (4MSOB), 2-propenyl glucosinolate (allyl), 4-hydroxy-indol-3-ylmethyl glucosinolate (4OHI3M), indol-3-ylmethyl glucosinolate (I3M), 4-methoxy-indol-3-ylmethyl glucosinolate (4MOI3M), and 1-methoxy-indol-3-ylmethyl glucosinolate (1MOI3M).

References Supplementary Methods

Brown P.D., Tokuhisa J.G., Reichelt M. & Gershenzon J. (2003) Variation of glucosinolate accumulation among different organs and developmental stages of *Arabidopsis thaliana*. *Phytochemistry* **62**, 471–481. https://doi.org/10.1016/S0031-9422(02)00549-6.

Burow M., Müller R., Gershenzon J. & Wittstock U. (2006) Altered Glucosinolate Hydrolysis in Genetically Engineered *Arabidopsis thaliana* and its Influence on the Larval Development of *Spodoptera littoralis*. *Journal of Chemical Ecology* **32**, 2333–2349. https://doi.org/10.1007/s10886-006-9149-1.

Karssemeijer P.N., de Kreek K.A., Gols R., Neequaye M., Reichelt M., Gershenzon J., van Loon J., & Dicke M. (2022) Specialist root herbivore modulates plant transcriptome and downregulates defensive secondary metabolites in a brassicaceous plant. *New Phytologist* **235**, 2378–2392. https://doi.org/10.1111/nph.18324.

Vadassery J., Reichelt M., Hause B., Gershenzon J., Boland W. & Mithöfer A. (2012) CML42-Mediated Calcium Signaling Coordinates Responses to *Spodoptera* Herbivory and Abiotic Stresses in Arabidopsis. *Plant Physiology* **159**, 1159–1175. https://doi.org/10.1104/pp.112.198150.
